# Supplementary material for: Endocytic Control of Cell‐Autonomous and Non‐Cell‐Autonomous Functions of p53
Source: Adv Sci (Weinh). 2026 Jan 30;13(30):e13765. doi: 10.1002/advs.202513765 (PMC13248759; doi:10.1002/advs.202513765)
Supplement: Supplementary file 1 — Supporting File 1: advs74070‐sup‐0001‐SuppMat.docx. [file ADVS-13-e13765-s001.docx]

**SUPPLEMENTARY INFORMATION**

**Endocytic control of cell-autonomous and non-cell-autonomous functions of p53**

Roberta Cacciatore^1‡^, Andrea Basile^1,2‡^, Stefano Freddi^1,2^, Irene Schiano Lomoriello^1,2^, Carlo Ribelle Zucca^1^, Giuseppe Ciossani^1^, Luigi Scietti^1^, Alessandro Cuomo^1^, Simona Ronzoni^1^, Simone Pelicci^1^, Mario Faretta^1^, Elena Zaccheroni^1^, Giuliana Pelicci^1,3^, Vittoria Matafora^4^, Angela Bachi^4^, Rosalind Helen Gunby^1^, Salvatore Pece^1,2^, Sara Sigismund^1,2^, Letizia Lanzetti^5,6^, Ivan Nicola Colaluca^1§^ and Pier Paolo Di Fiore^1,2§^*

^1^ IEO, European Institute of Oncology IRCCS, Milan, Italy

^2^ Department of Oncology and Haemato-Oncology, University of Milan, Milan, Italy

^3^ Department of Translational Medicine, University of Piemonte Orientale, Novara, Italy

^4^ IFOM ETS, the AIRC Institute of Molecular Oncology, Milan, Italy

^5^ Department of Oncology, University of Torino Medical School, Torino, Italy

^6^ Candiolo Cancer Institute, FPO - IRCCS, Candiolo, Torino, Italy

**Figure S1. Additional data to Figure 1 of the main text. A.** Label-free quantification (LFQ) values of proteins identified in the NUMB-1 and NUMB-2 interactomes. NUMB-1/2–specific interactors are indicated in red, while those interacting with all isoforms are indicated in blue. Hits from the mass spec analysis were depleted of proteins present in the so-called “crapome” – a list of proteins frequently detected as non-specific contaminants in affinity purification mass spectrometry experiments [http://www.crapome.org/].^[1]^ The efficiency of the co-IP and mass spec analysis is supported by the detection of several known NUMB interactors (all isoforms), such as various AP2 adaptor complex subunits or EH-domain-containing proteins (EPS15, EPS15L1), indicated in the graph. **B,C.** MCF10A (**B**) or HBL-100 (**C**) cell lysates were IP with anti-NUMB and IB as indicated (right). A peptide corresponding to the NUMB epitope was used as negative control to compete antibody binding during the IP; l.e., long exposure. **D.** HEK-293 cells were silenced with NUMB siRNA (+) or Ctrl siRNA (-) and transfected with SNX9-HA as shown (top). Anti-HA IPs were IB as shown. An HA peptide was used as negative control to compete antibody binding during the IP. **E.** HEK-293 cells transfected with NUMB-1-FLAG were silenced with SNX9 siRNA (+) or Ctrl siRNA (-) and transfected with a siRNA-resistant SNX9-HA (s.r.) as shown (top). Anti-FLAG IPs were IB as shown (right). A FLAG peptide was used as negative control to compete antibody binding during the IP.


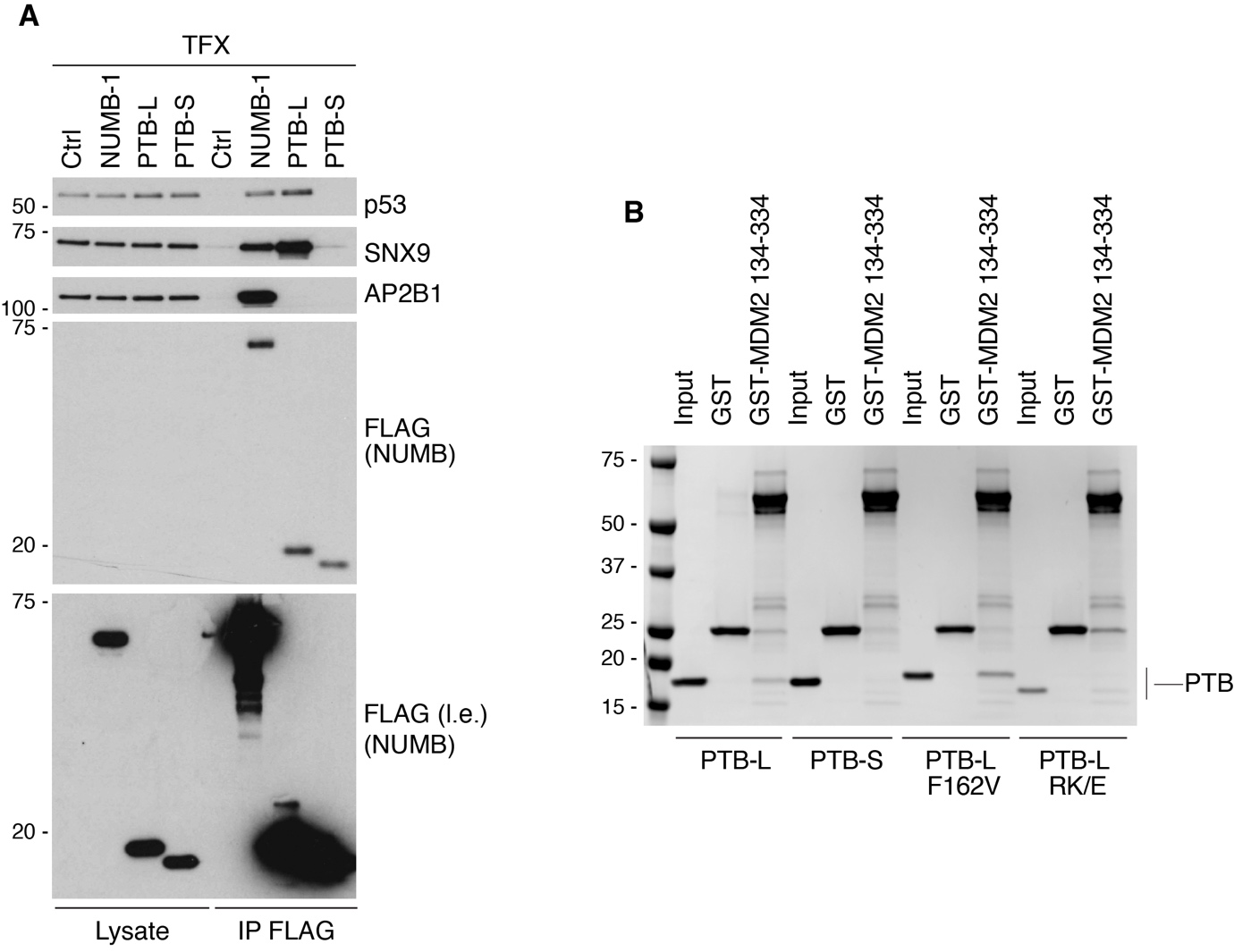


**Figure S2. Additional data to Figures 2A and B of the main text. A.** The same blots as in Figure 2A are shown, together with a long exposure (l.e.) of the anti-FLAG (NUMB) IB to allow visualization of the FLAG-tagged NUMB constructs in the total cellular lysate. **B.** *In vitro* binding assay performed with purified GST-MDM2 fragment 134-334 (containing the NUMB-interacting region^[2]^) or GST alone, immobilized on GSH beads, and the indicated purified NUMB-PTB proteins. Bound proteins were detected by Coomassie staining.


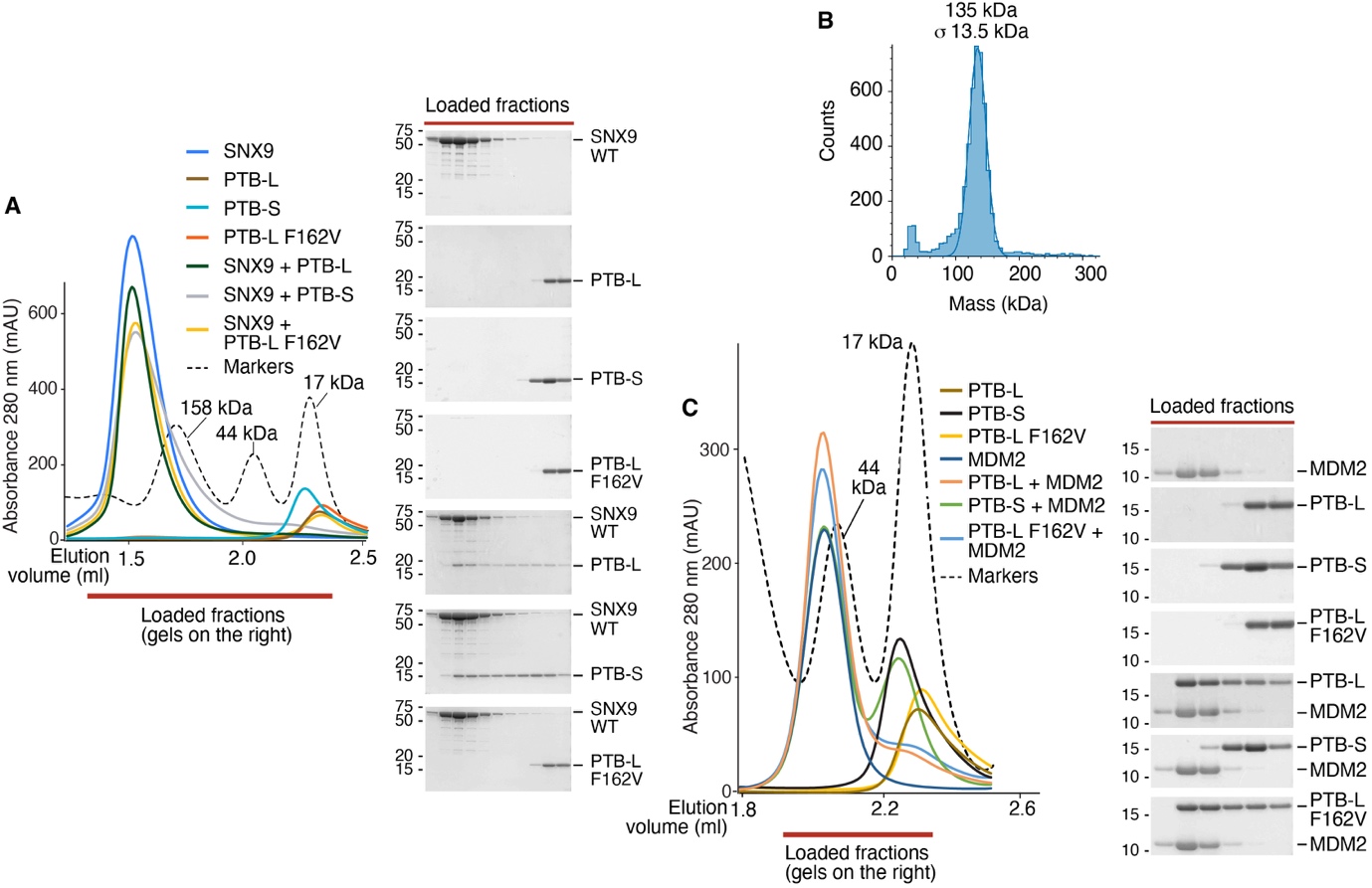


**Figure S3. Additional data to Figure 2C and 2D of the main text. A.** Left, size-exclusion chromatography (SEC) elution profiles of the indicated purified proteins, alone or in combination. The red bar below the x-axis indicates the fractions loaded onto the gels shown on the right. Right, aliquots of the collected fractions were resolved by SDS-PAGE and proteins were detected by Coomassie staining. Note that the shift towards the left of PTB-L and PTB-S when combined with SNX9, compared with PTB-L and PTB-S alone, is indicative of the formation of a complex. This shift is not observed with the F162V mutant. Prior to SEC analysis, we observed minimal precipitation upon mixing certain species of SNX9 and PTB. This precipitate was removed by centrifugation of the sample before injection. This phenomenon may account for the slight reduction in peak absorbance observed for some SNX9/PTB complexes compared to SNX9 alone. The elution profile of the molecular weight marker shown here is consistent across all SEC experiments presented in the manuscript, as the same chromatography column was used throughout. **B.** As shown in panel A, SNX9 elutes in SEC at an apparent molecular weight above 158 kDa. To investigate this behavior, we analyzed purified full-length SNX9 by mass photometry. The main species detected had an average molecular mass of approximately 135 kDa, consistent with the formation of a SNX9 homodimer (monomer MW: 66.5 kDa), as expected given the dimerization properties of the SNX9 BAR domain.^[3]^ σ 13.5 kDa represents the standard deviation. The slight discrepancy between the SEC elution profile and the calculated dimer mass may be attributed to the native conformation of SNX9, which can influence its hydrodynamic behavior. The y-axis of the graph shows the number of molecules analyzed (counts). **C.** SEC elution profiles of the indicated combinations of purified proteins (left). Fractions, indicated by the red bar, were analyzed SDS-PAGE and Coomassie staining (right). MDM2 corresponds to the MDM2 fragment aa 216-302, harboring phosphomimetic mutations which increase binding to NUMB.^[2]^ The data confirm that the interaction between MDM2 and NUMB-PTB is largely dependent on the Ex3-encoded sequence of NUMB. Note that the MDM2^216-302^ fragment alone eluted around the 44-kDa MW marker, as did the PTB-L/MDM2^216-302^ complex. This is likely due to the intrinsically disordered nature of the MDM2^216-302^ region, as previously reported,^[2]^ which causes the monomer to elute in SEC at an apparent molecular weight significantly higher than its theoretical mass. Some of the gels and SEC elution profiles of panel A are replicated in panel C (PTB proteins alone), because the two experiments were performed as a single experiment but are shown separately to avoid overcrowding of the tracks in the SEC elution profiles.


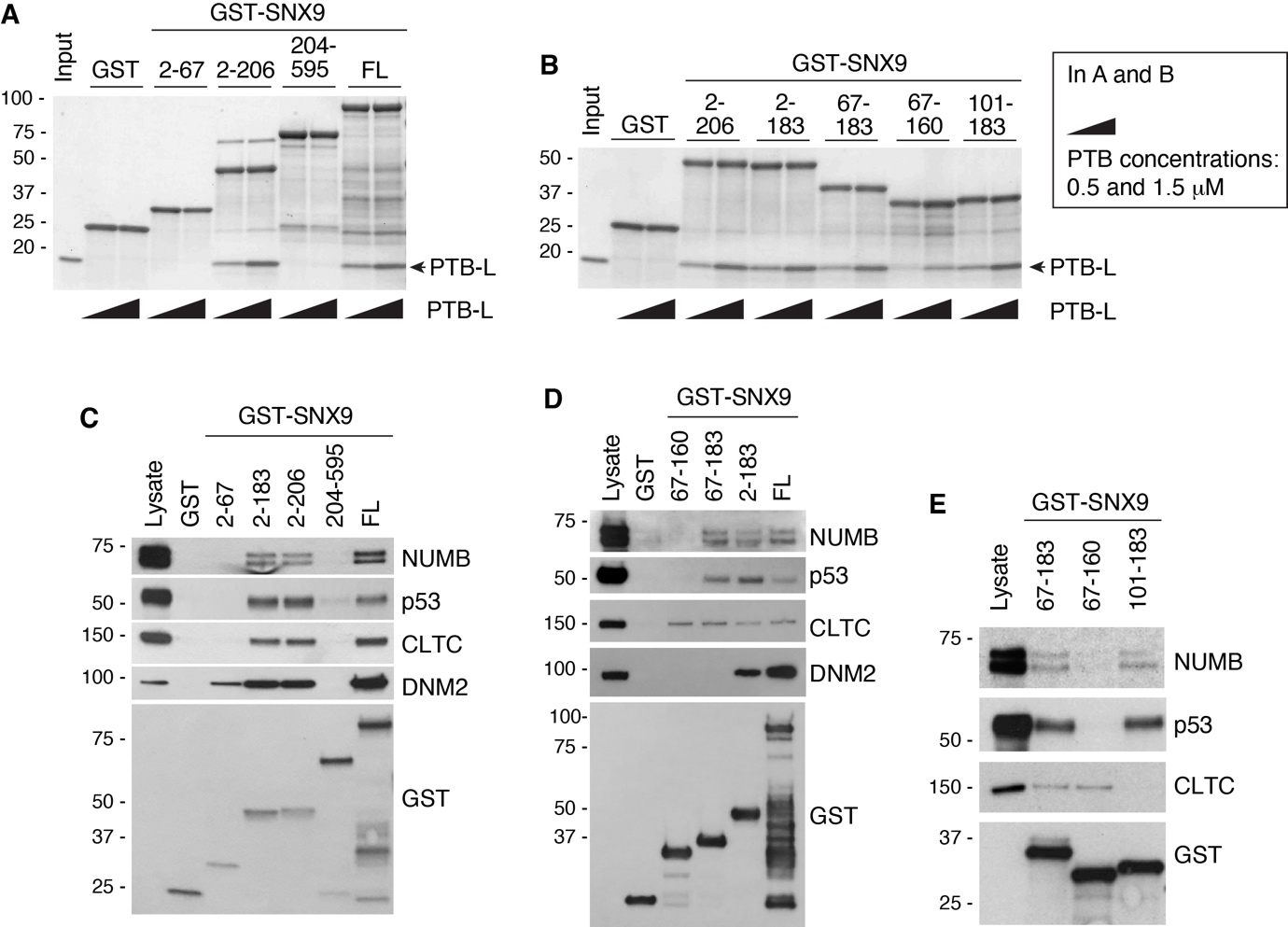


**Figure S4. Additional data to Figure 2E of the main text: identification of the SNX9 LC domain as the binding surface for NUMB and p53. A, B.** *In vitro* binding assays with purified GST-SNX9 fragments or full-length (FL) protein immobilized onto GSH beads and increasing amounts of purified PTB-L (indicated by triangles). Bound proteins were detected by Coomassie staining. **C-E.** Pull-down assays performed with HEK-293 cell lysates and purified GST-SNX9 fragments or FL protein immobilized onto GSH beads, followed by IB as indicated on the right. DNM2 (dynamin 2) and CLTC (clathrin heavy chain) are known SNX9 interactors (see Figure 2E), used here to confirm that the engineered SNX9 fragments display the expected binding abilities.


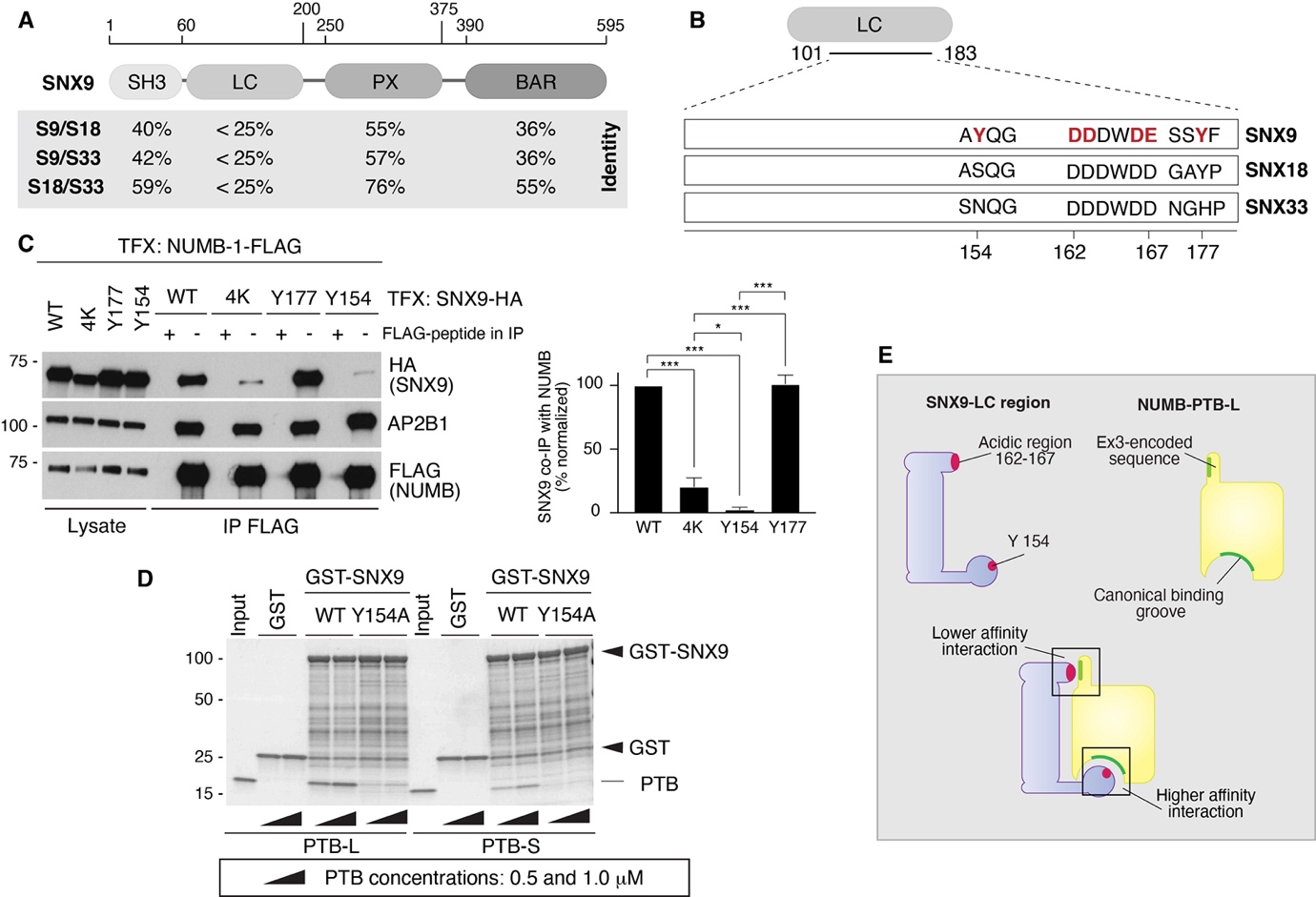


**Figure S5. Additional data to Figure 2E of the main text: fine mapping of the SNX9 LC domain and deconvolution of the bidentate interaction with NUMB-PTB-L. A.** Domain organization of SNX9, SNX18 and SNX33 and sequence identities of the indicated domains, as published.^[4]^ **B.** Alignment of the regions spanning residues 101 – 183 within the LC domains of SNX9, SNX18 and SNX33. Since SNX18 and SNX33 do not bind to NUMB-PTB-L (Figure 1H), and since PTBs display preference for Y-based motifs for binding to their canonical binding groove,^[5]^ we reasoned that Y154 of SNX9, not present in SNX18 and SNX33, could be a candidate binding site. In addition, Y177 present in SNX9 and SNX18 but not in SNX33 could also be a candidate. To investigate the involvement of these Y residues (in red), we mutagenized them to A. The positions of the four acidic amino acids (D and E in red) mutagenized to K in the SNX9 4K mutant shown in panel **C** (see also main text) are also indicated. **C.** Left, HEK-293 cells were transfected (TFX) with NUMB-1-FLAG along with the indicated SNX9-HA constructs (WT or 4K, Y177 and Y154 mutants), followed by anti-FLAG IP and IB as shown (right). A FLAG peptide was used as negative control to compete with antibody binding during the IP Mut. 4K: SNX9 D162, D163, D166, E167>K. Mut. Y154: SNX9 Y154>A. Mut. Y177: SNX9 Y177>A. Right, quantitation of the amount of SNX9-HA (WT or mutant) binding to NUMB-1-FLAG by densitometry analysis of three independent co-IP experiments. The amount of SNX9 that co-IPs with NUMB was normalized to the level of expression of each SNX9-HA construct (see “lysate” lanes in the left panel) and expressed as a percentage of the co-IP between WT-SNX9 and NUMB. Results are presented as the mean ± SD. *, P <0.05; ***, P < 0.001. **D.** *In vitro* binding assay performed with purified full-length GST-SNX9 wild type (WT) or mutant GST-SNX9-Y154A immobilized on GSH beads and increasing amounts (0.5 and 1.0 μM) of NUMB-PTB-L or NUMB-PTB-S (indicated by triangles). Bound proteins were detected by Coomassie staining. **E.** Model of the bidentate interaction between the SNX9 LC domain and NUMB PTB-L. The SNX9-Y154 residue mediates a higher affinity interaction with the PTB-binding groove. A lower affinity interaction is mediated by the acidic region of the SNX9 LC domain and the Ex3-encoded sequence of PTB-L.


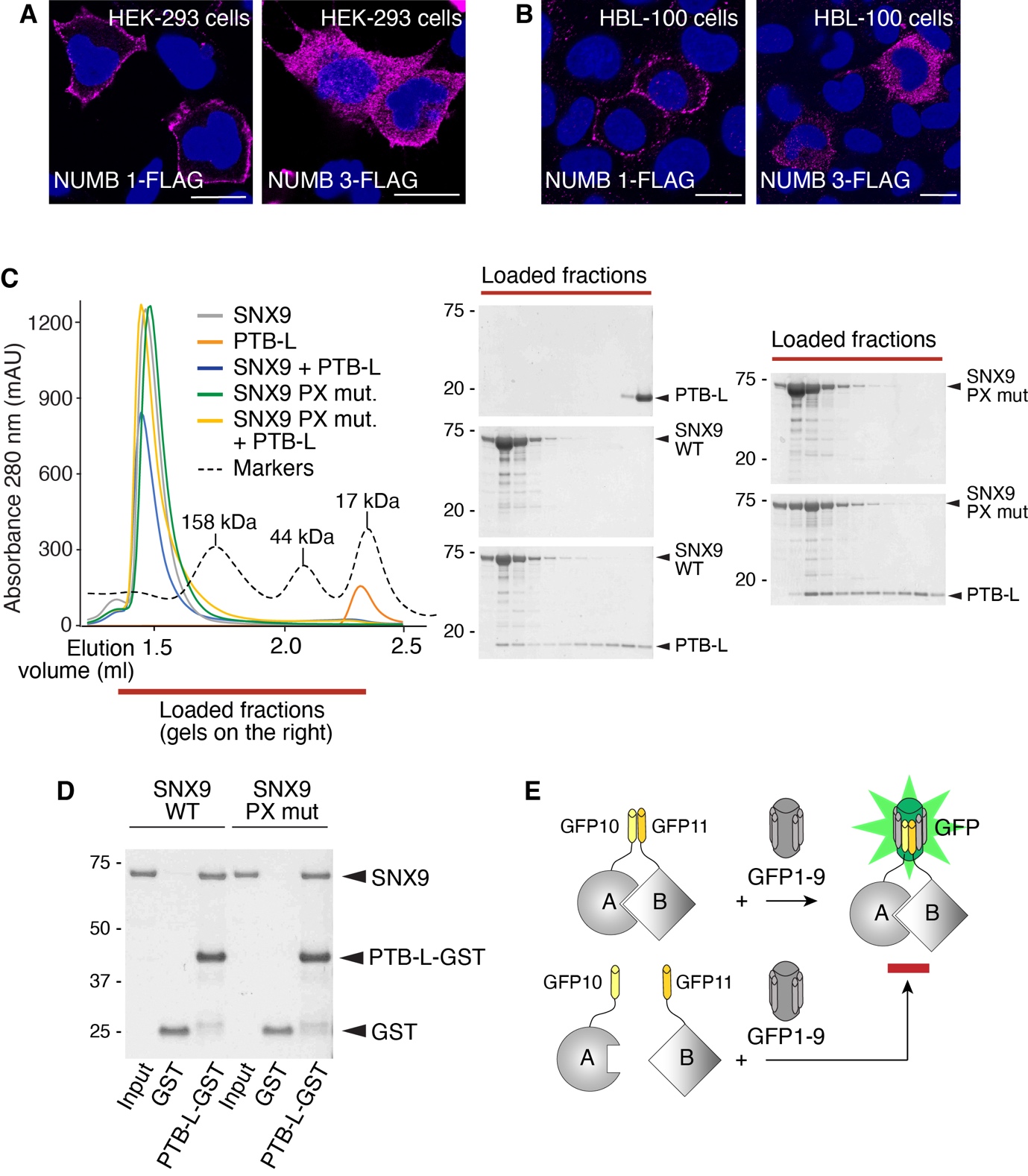
**Figure S6. Additional data to Figure 3 of the main text. A, B.** HEK-293 cells (**A**) or HBL-100 cells (**B**) were transfected with FLAG-tagged NUMB-1 or NUMB-3. Transfected NUMB was visualized by IF imaging with an anti-FLAG antibody (purple). Blue, DAPI counterstain. Bar, 20 µm. **C.** Size-exclusion chromatography (SEC) elution profiles of the indicated combinations of purified proteins (left). Fractions, indicated by the red bar, were analyzed by SDS-PAGE and Coomassie staining (right). **D.** *In vitro* binding assay with purified NUMB-PTB-L-GST or GST alone, immobilized on GSH beads, and purified SNX9-WT or SNX9-PXmut proteins. Bound proteins were detected by Coomassie staining. **E.** Scheme of the tripartite SPLIT-GFP system. Tripartite GFP complementation relies on the reconstitution of fluorescence through the interaction of three GFP fragments. Proteins of interest (A and B in the scheme) are tagged individually with two short fragments of GFP, GFP10 and GFP11. When A and B physically interact, GFP10 and GFP11 can self-assemble with a larger detector fragment, GFP1-9, expressed in the cell, enabling proper GFP folding and chromophore maturation (top). The resulting fluorescence allows the precise detection of protein-protein interactions or colocalization within the cell with minimal background signal. When A and B fail to interact the GFP fluorescence signal is not emitted (bottom).


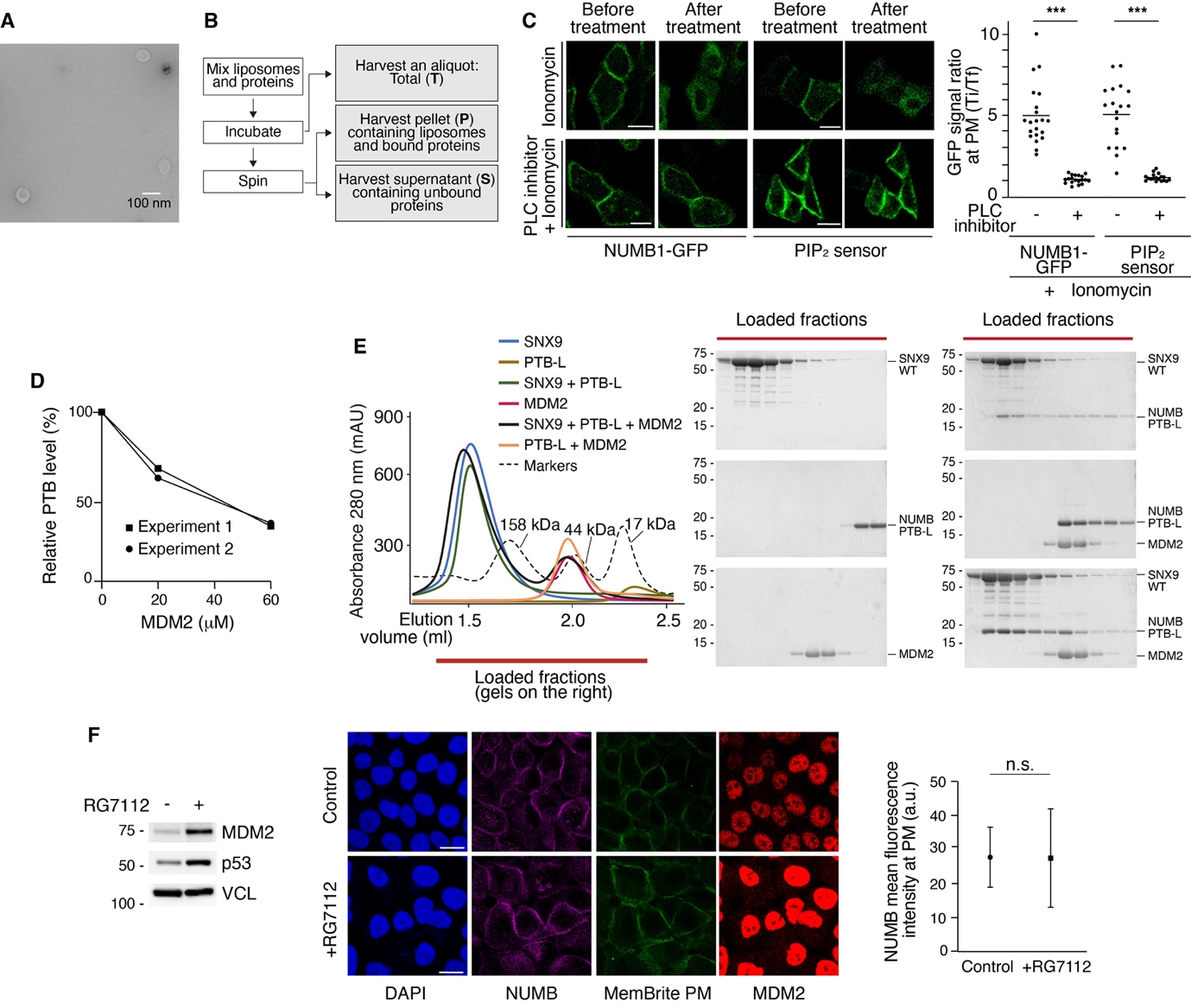


**Figure S7. Additional data to Figure 4 of the main text. A.** Representative transmission electron microscopy images of liposomes used in the liposome binding assays described in the main text. Bar, 100 nm. **B.** Scheme of the liposome binding assay. **C.** MCF10A cells were transfected with either NUMB1-GFP or a PIP_2_ sensor-YFP construct, and treated as indicated. The PIP_2_-YFP sensor comprised the PH domain of PLCδ1 fused to YFP; it predominantly binds plasma membrane PIP₂ and enables real-time visualization of its localization in living cells.^[6]^ Where indicated, cells were treated with the PLC inhibitor U73122 (10 µM) or DMSO control for 3 min before adding ionomycin (10 µM) for 6 min. Left. Representative GFP or YFP epifluorescence (green) images from time-lapse microscopy before and after treatment. The fluorescence signal is displayed using the same color scale for all samples, as images were acquired using identical excitation and emission wavelengths compatible with both fluorophores. Bar 20 µm. Right. Quantitation of the experiment. For each cell analyzed, the green signal at the PM was quantified at the beginning (Ti) and end of treatment (Tf). The average Ti/Tf ratio for each analyzed cell was plotted as an individual data point. Results are from two independent experiments, with the mean for each condition indicated by the horizontal line. ***, p <0.001. **D.** Quantitation of the experiment in Figure 4B, related to the PTB-L/MDM2^216-302^ combination, repeated on two independent experiments. **E.** Left, size-exclusion chromatography (SEC) elution profiles of the indicated combinations of purified proteins. Fractions, indicated by the red bar were analyzed by SDS-PAGE and Coomassie staining (right). Note that the PTB-L profiles shifts to the left when combined with SNX9 or MDM2^(216-302)^ compared with PTB-L alone, indicating the formation of dimeric complexes. When all three proteins are present, a tripartite complex is not formed; rather PTB-L forms independent dimeric complexes with SNX9 and MDM2^(216-302)^. This finding is supported by the fact that MDM2^(216-302)^ is absent in the fractions enriched in the PTB-L:SNX9 complex. The gels and SEC elution profiles (with the exception of the combination PTB-L:SNX9:MDM2) are replicated from Figure S3A,C because the experiments were performed as a single experiment but are shown separately to avoid overcrowding of the tracks in the SEC elution profiles. **F.** Left, MCF10A cells were treated with RG7112 (5 μM, 6 h), followed by IB with the indicate antibodies (Vinculin, VCL, is used as a loading control). By inhibiting the action of MDM2 on p53, RG7112 causes an increase in the levels of p53, which in turn stimulate transcription of the MD2 gene.^[7]^ Middle, cells, treated as in left, were incubated with MemBrite Fix 488/515 (Biotium) according to the manufacturer’s instructions, fixed, and processed for IF for endogenous NUMB (purple) and MDM2 (red). The plasma membrane (PM) was visualized with MemBrite (green), and nuclei were counterstained with DAPI (blue). Scale bar, 20 μm. Right, ten random fields of view per condition (∼10 cells per field) were acquired. The MemBrite 488 signal was used to generate a PM mask in each field, and NUMB fluorescence intensity at the PM was measured within this region. The mean NUMB fluorescence intensity at the PM was then obtained by averaging across the ten fields. n.s.: not significant.


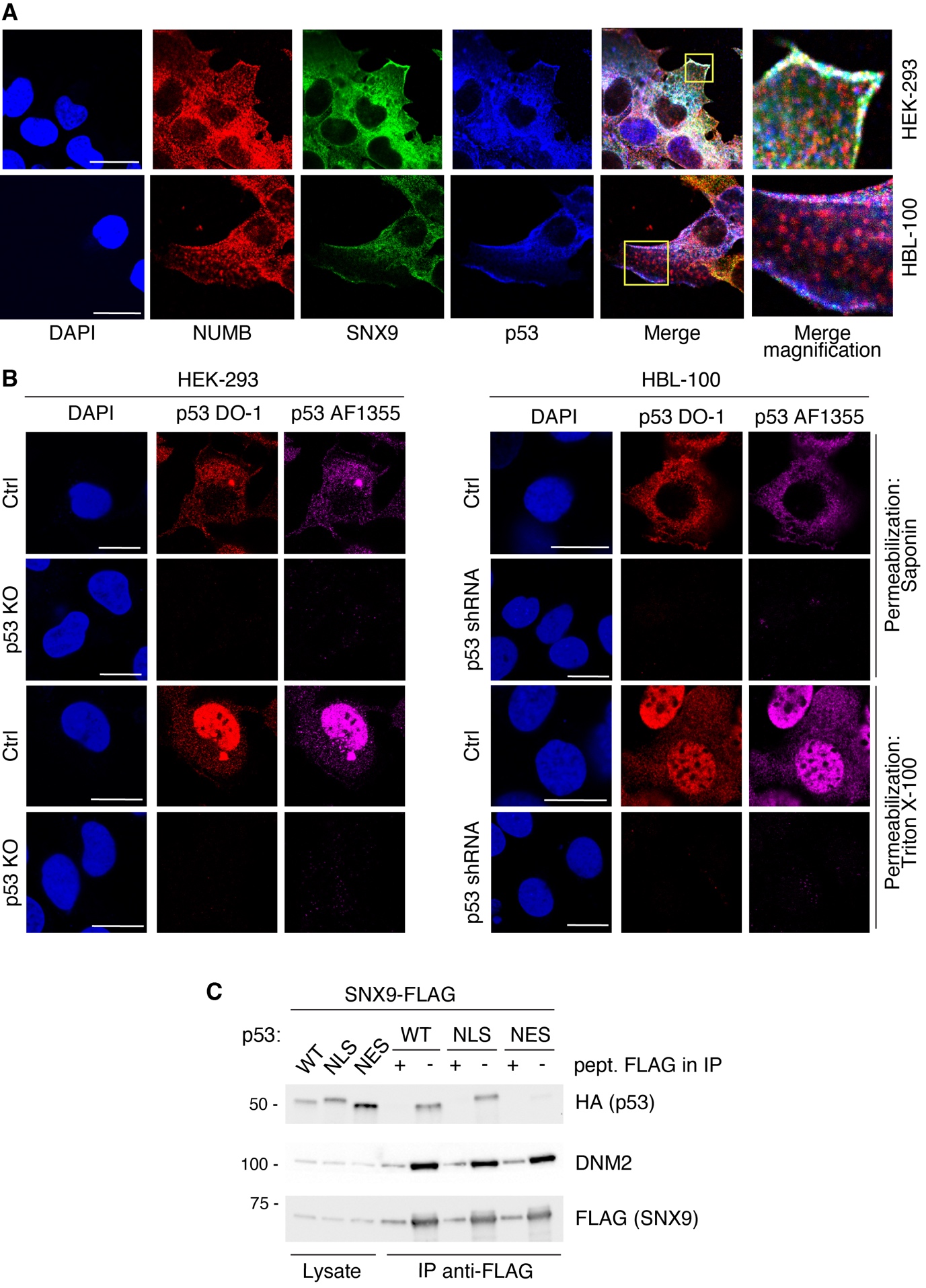


**Figure S8. Additional data to Figure 5 of the main text. A.** IF analysis of HEK-293 cells (top) and HBL-100 cells (bottom) with anti-NUMB (red), anti-SNX9 (green), and anti-p53 (blue) antibodies. Cell permeabilization was performed with saponin to permeabilize preferentially the PM, allowing for a clearer assessment of cytoplasmic and PM localized p53 by minimizing the nuclear signal. This approach was necessary due to the significantly higher levels of nuclear p53 compared to cytoplasmic levels. Merge with magnification of boxed areas is shown on the right. DAPI, nuclear stain. Bar, 20 µm. **B.** The panel shows a series of controls to illustrate the specificity of the staining obtained with the two anti-p53 Abs used in the study (DO-1 and AF1355, stained as indicated on top) and the different effects of solubilization with saponin or Triton X-100 (upper panels and lower panels). Specificity was shown in HEK-293 (left panels) and HBL-100 (right panels) cells by ablating p53 expression either by KO (in HEK-293) of by shRNA (in HBL-100). As shown, the permeabilization with saponin, which leaves the nuclear membrane largely intact, allows for better appreciation of the non-nuclear pool of p53, which is predominant in the cell, as shown by permeabilizing the cells with Triton X-100. DAPI, nuclear counterstain. Bar, 20 µm. **C.** HEK293 p53 KO cells were transfected with SNX9-FLAG and the indicated HA-tagged p53 constructs, expressed under a minimal CMV promoter to maintain p53 at near-physiological levels. Anti-FLAG IP were analyzed by IB as shown. A FLAG peptide was used as a negative control to compete antibody binding during the IP. DNM2 was included as a positive control for SNX9 binding. The p53 NLS mutant carries alanine substitutions at residues K305, R306, K319, K320, and K321. The p53 NES mutant lacks the region encompassing residues 340–355. The NLS and NES of p53 are as described.^[8-9]^

**
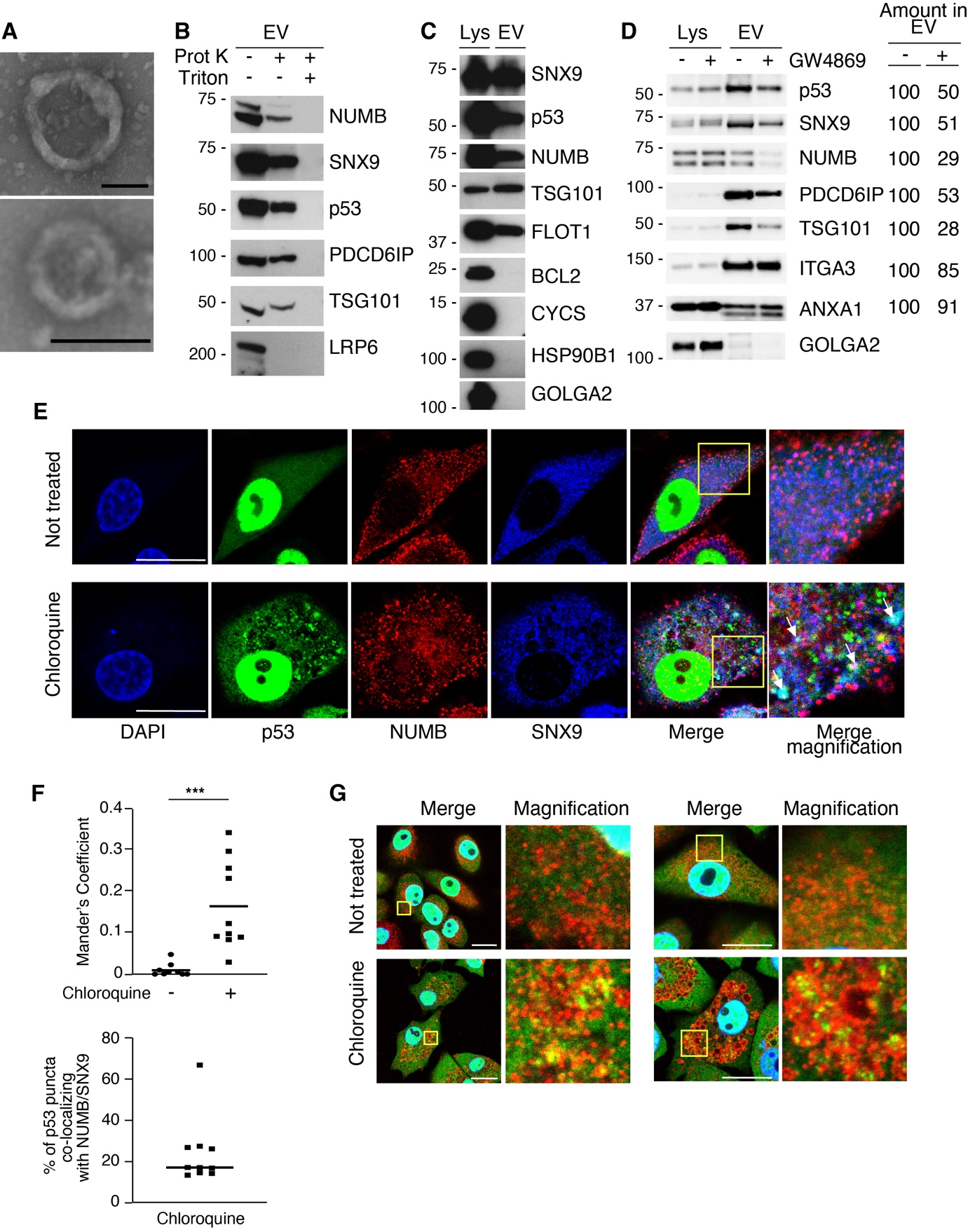
**

**
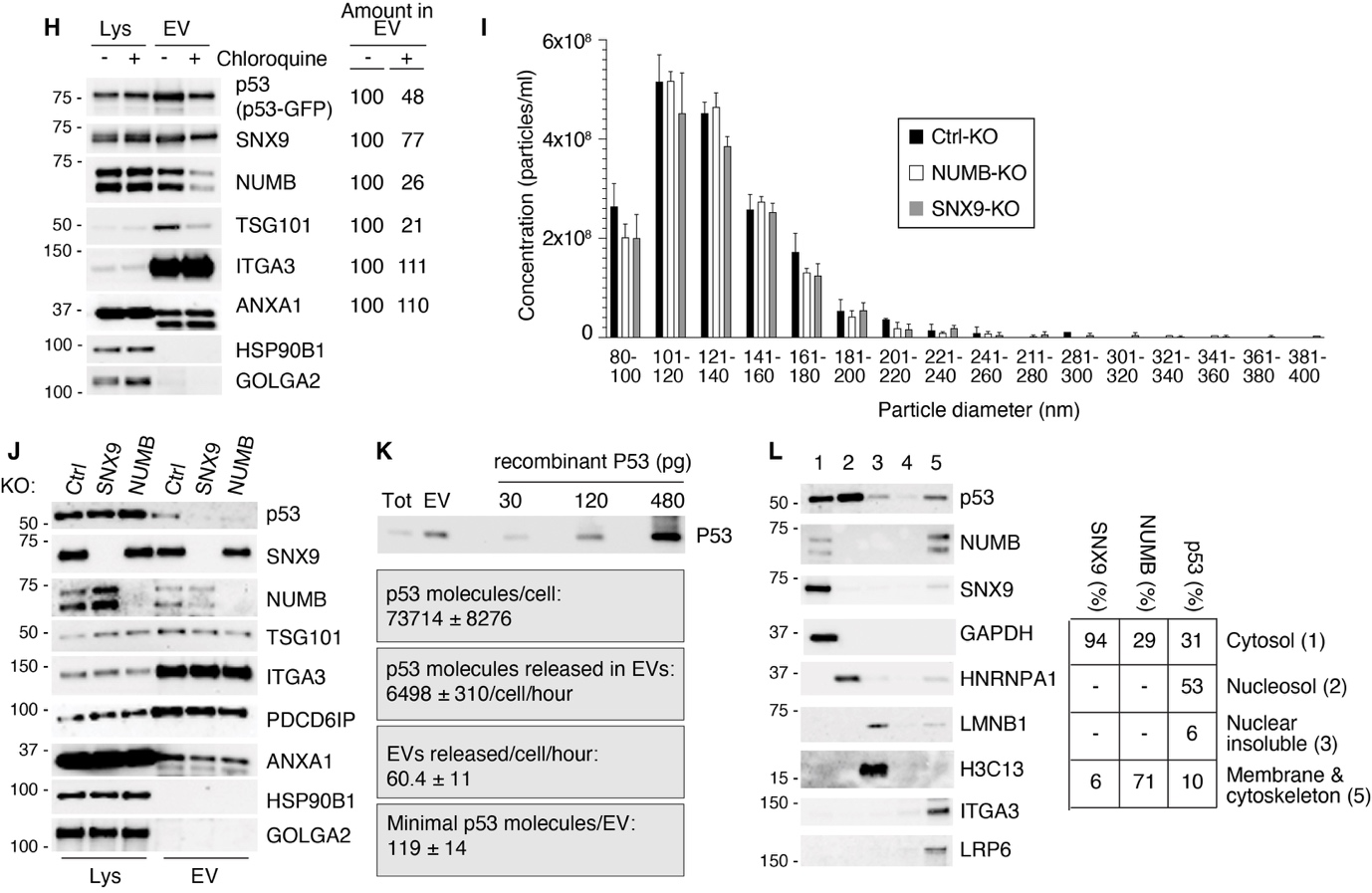
**

**Figure S9. Additional data to Figure 6 of the main text. A**. The quality of the EV purification was controlled by visualizing vesicles using electron transmission microscopy. Representative images of EVs purified from the conditioned medium of HEK-293 cells are shown; bar 100 nm. **B.** To demonstrate that proteins present in EVs preparations are protected by a phospholipid bilayer, we treated them with Proteinase K (Prot K). EVs purified as in ‘A’ were treated with Prot K (5 μg/ml) and Triton X-100 (1% final) as indicated and IB as shown on the right. **The partial decrease in the protein levels in EVs following Prot K treatment** may be caused by multiple factors, as previously reported:^[10-11]^ (i) the high shear stress generated during resuspension of the ultracentrifugation pellet could result in partial vesicle disruption; and (ii) cleavage of membrane-associated EV proteins might compromise vesicle integrity, leading to increased susceptibility to proteolytic degradation. **C.** In the experiments shown in Figure 6A, 6C, 6G, S9A, S9B, S9I we used EVs purified from HEK-293 cells. In the experiments shown here and in Figure 6B, 8A, 8B, and S11B, we used EVs purified from Expi293 cells, a derivative of HEK-293 cells that grows in suspension at high density, thus allowing a higher yield of purified EVs. We demonstrated that EVs derived from Expi293 cells show the same characteristics as those derived from HEK-293 cells, especially in terms of the amount of SNX9, NUMB, p53 and other markers packaged into EVs, as shown by IB (compare with Figure 6A). Lys, total cellular lysate. **D.** MCF10A cells were treated with GW4869 (20 μM for 24 h). EVs were purified from conditioned medium and analyzed by IB as indicated (right). **E.** MCF10A cells stably expressing p53–GFP (green) were treated with chloroquine (50 µM, 24 h) or vehicle control and subjected to IF staining for NUMB (red) and SNX9 (blue, fourth panel). DAPI staining is shown in blue. Merged and magnified images are shown on the right. Scale bar, 20 µm. **F.** Top, quantification of NUMB-SNX9 and p53-GFP co-localization in the experiment shown in panel E, performed using Manders’ coefficient. Ten random fields per condition were analyzed by confocal microscopy. Co-localization analysis was carried out in two steps: (i) the NUMB–SNX9 co-cluster area (defined as the region where both signals overlap) was identified, and (ii) the Manders’ coefficient between this area and the p53–GFP signal was calculated. Results are presented as individual points with their mean (line). ***, p<0.001. Bottom, the same fields analyzed in the upper panel (chloroquine-treated cells only) were further evaluated for the percentage of p53-GFP puncta co-localizing with NUMB-SNX9. p53-GFP puncta were defined as objects with an area greater than 0.05 µm², and the percentage of p53-GFP puncta containing a NUMB-SNX9 co-cluster area (as previously defined) was measured. Results are presented as individual points with their mean (line). **G.** Additional microscopic fields from the experiment shown in Figure 6D are displayed (merge and magnification panels only). p53-GFP (green), RAB7 (red), DAPI (blue). Bar, 20 μm. **H.** EVs were purified from the conditioned medium of MCF10A cells stably expressing p53-GFP and IB as indicated. On the right, a densitometric quantification of the signals of the various proteins in the EV fractions is shown, in arbitrary units. **I.** EVs from HEK-293 Ctrl-KO, SNX9-KO and NUMB-KO cells, as described in Figure 6G, were analyzed by tunable resistive pulse sensing (TRPS) to measure concentration and diameter. Results are expressed as means ± SD of three technical replicates. The one-way ANOVA test was not significant, indicating that the KO of SNX9 or NUMB does not affect the number or size of EVs. **J.** EVs were purified from the conditioned medium of Ctrl-KO, SNX9-KO or NUMB-KO U2OS cells, as indicated on top, and IB as shown (right). **K.** MCF10A cells were cultivated to near confluence (12 x 10^6^ cells/15 cm plate), washed three times with PBS, and re-incubated in serum-free medium for 20 min. The EVs released in this 20 min window were purified from the conditioned medium. Purified EVs (1/13^th^ of total preparation) was analyzed IB as indicated (EV lane), alongside total cellular lysate (1/2380^th^ of total lysate) and the indicated amounts of purified recombinant p53. Densitometry analysis of two independent experiments was performed to quantify the amount of p53 present in cells and EVs. Bottom, the number of p53 molecules/cell, number of p53 molecules released in EVs/cell/h, number of EVs produced by a cell/hour, and the minimal number of p53 molecules present in a single EV are shown (mean ± SD). **L.** IB analysis of subcellular fractions obtained from MCF10A cells (see Experimental section for details of the fractionation procedure). The fractions were loaded after normalization to their respective volumes to ensure proper comparison. 1, cytosol; 2, nucleosol; 3, insoluble nuclear components (chromatin and nuclear lamina); 4, intermediate fractions within the gradient, containing minor material corresponding to the transition between fractions 3 and 5; 5, membranes and cytoskeletal components. To evaluate the quality of the fractionation, we used well-established compartmental markers: GAPDH for the cytosol, HNRNPA1 for the nucleosol, LMNB1 (Lamin B1) and H3C13 (Histone H3) for insoluble nuclear material (lamina and chromatin, respectively), and LRP6 and ITGA3 for the plasma membrane. Densitometric quantification of each fraction (right) revealed that NUMB is predominantly associated with membranes compared to the soluble cytoplasmic pool, SNX9 is mainly distributed in the cytosol with only a minor membrane-associated fraction, and p53 exhibits a heterogeneous distribution – consistent with its multiple cellular functions – with the majority located in the nucleus and approximately 40% detected in the cytosolic and membrane/cytoskeletal fractions.


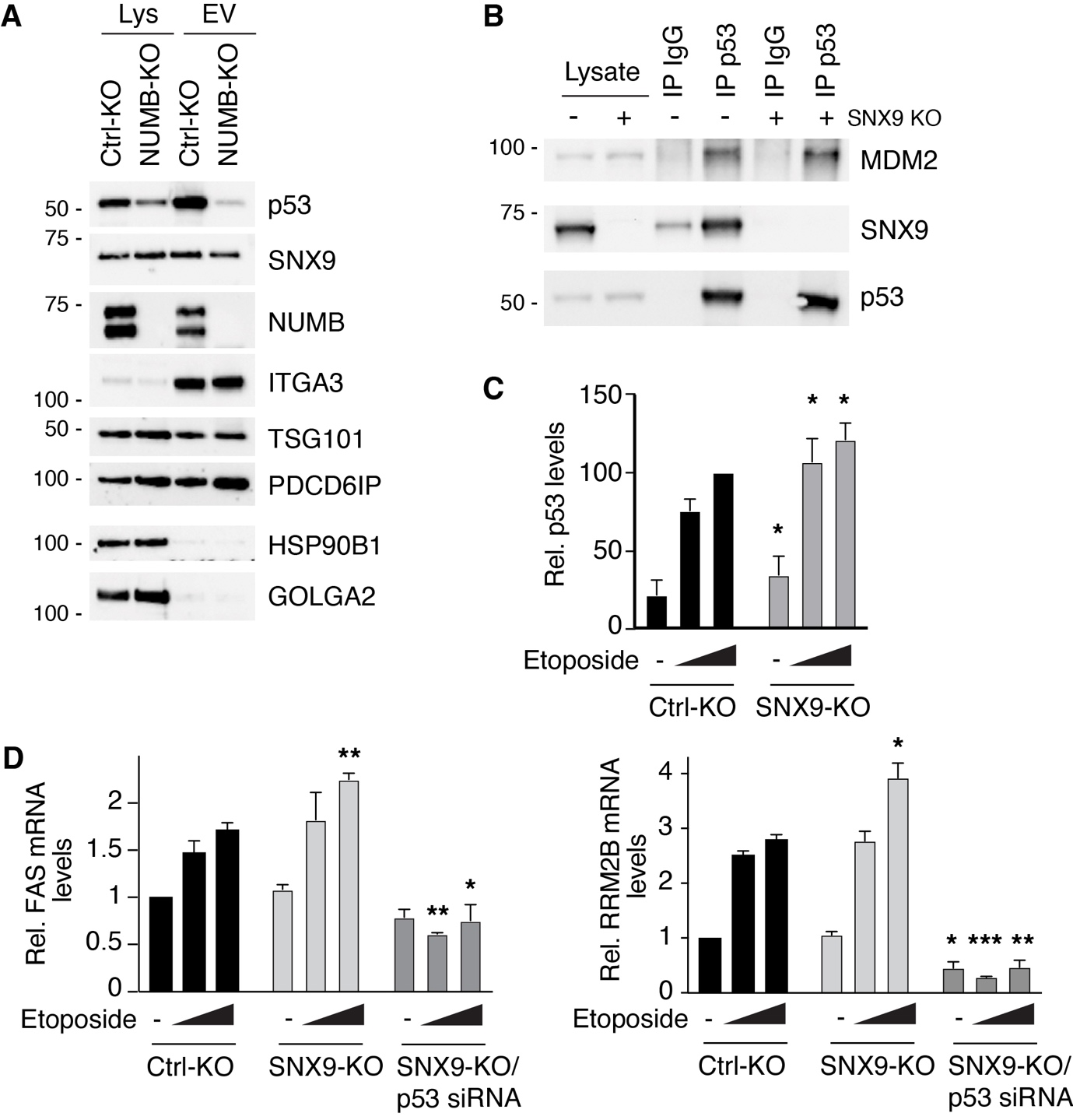


**Figure S10. Additional data to Figure 7 of the main text. A.** We performed NUMB-KO in MCF10A cells. Also in this case, as in the case of MCF10A-SNX9-KO cells (Figure 7A), p53 levels in EVs were reduced compared with Ctrl-KO cells. Notably, basal intracellular levels of p53 were increased following SNX9-KO (Figure 7A and subsequent Figure S10C); conversely, in MCF10A-NUMB-KO cells, the levels of intracellular p53 were decreased, as expected due to the inhibitory action of NUMB on MDM2.^[12]^ Thus, we pursued our biological analysis of cell-autonomous effects of inhibition of p53 release through exosomes on MCF10A-SNX9-KO cells (shown in Figure 7), to avoid the confounding effects of the NUMB dual/opposite action on p53 levels. When comparing the effects of NUMB-KO in HEK-293 (Figure 6G) and MCF10A cells (this panel) there are some interesting differences to be commented upon. In particular, NUMB silencing in HEK-293 does not reduce p53 protein levels (Figure 6G). This is **likely attributable** to the presence of stably integrated adenoviral E1A and E1B gene products, which modulate p53 dynamics. E1A promotes p53 expression, while E1B inhibits p53-mediated apoptosis and cell cycle arrest, collectively leading to stabilization of p53 protein levels despite NUMB loss.^[13-14]^ **B.** We examined the interaction between p53 and MDM2 following SNX9 ablation by performing a co-immunoprecipitation (co-IP) assay, in which endogenous p53 was immunoprecipitated from SNX9 KO and Ctrl-KO cells. Cells were also treated with the proteasome inhibitor MG132 (5 μM) for 3 hours to stabilize MDM2 and facilitate its detection. The results revealed a comparable level of interaction between p53 and MDM2 in both cell types. This suggests that the pool of p53 accumulating as a result of its reduced association with SNX9 is not further sequestered by MDM2, consistent with the overall increase in intracellular p53 levels observed in Figure 7A,B. MCF10A SNX9 KO and Ctrl-KO cell lysates were IP using an anti-p53 antibody or irrelevant IgGs and IB as indicated. **C.** p53 levels from Figure 7B and three additional independent biological replicates were quantified by densitometric analysis using the Fiji software. Values are shown normalized to Ctrl-KO cells treated with 50 μM etoposide (=100). Values are expressed as mean ± SD. **D**. In Figure 7C, we analyzed the levels of MDM2 and CDKN1A mRNAs, two of the best p53-target genes. We also examined, and display here, two additional well-characterized p53 transcriptional targets, FAS (left) and RRM2B (right),^[15-16]^ whose expression in MCF10A cells was confirmed to be p53-dependent (this panel). Both genes showed a trend toward higher expression in SNX9 KO cells following etoposide treatment, as assessed by qPCR. However, a statistically significant increase was only observed at the highest etoposide concentration when data from three independent biological replicates were combined. This may reflect the generally lower transcriptional activation of FAS and RRM2B compared to MDM2 and CDKN1A, making subtle changes more difficult to detect. In the various panels, *, p<0.05; **, p<0.01, ***, p<0.001 *vs*. corresponding Ctrl-KO sample.

**
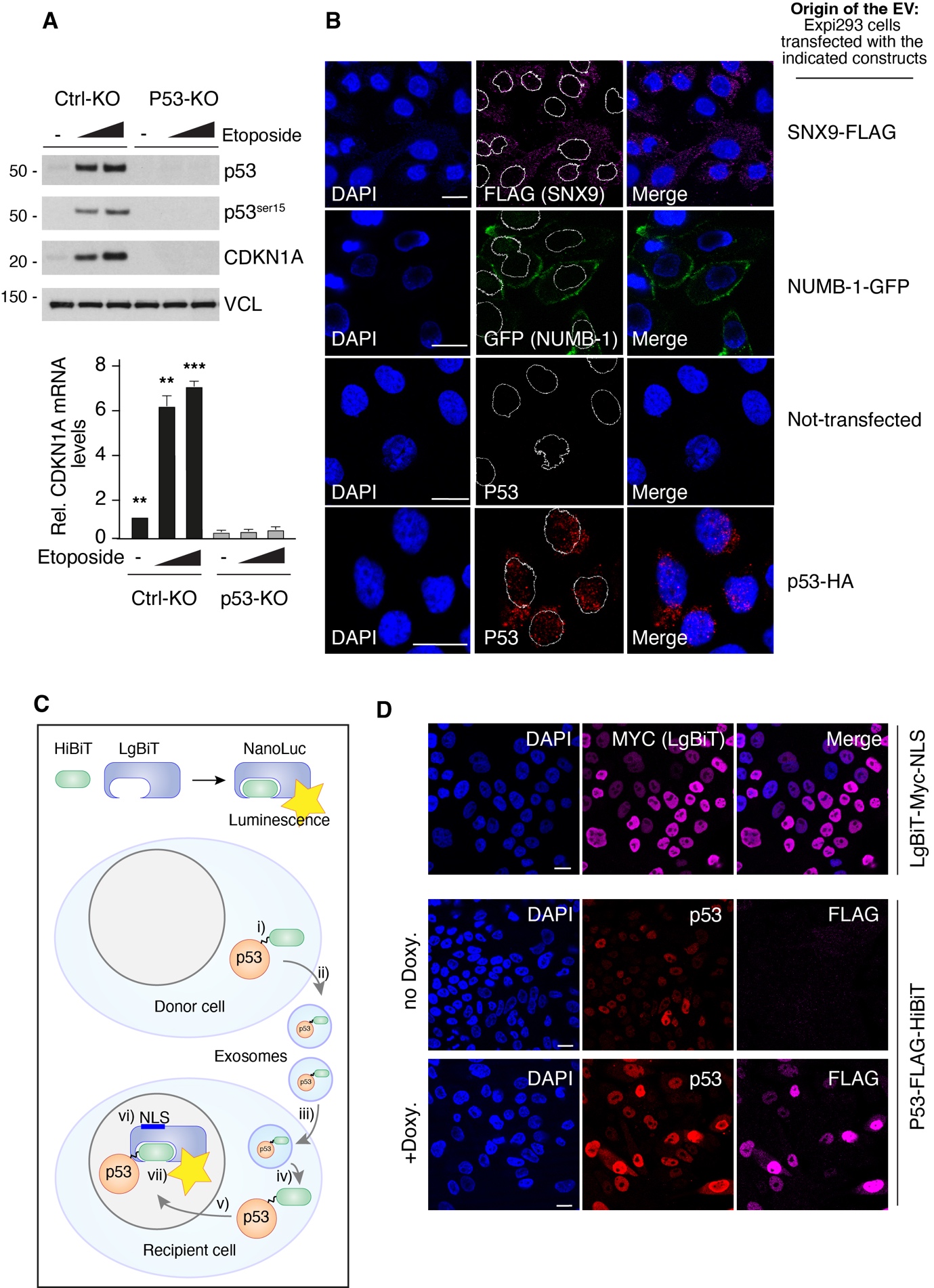
**

**
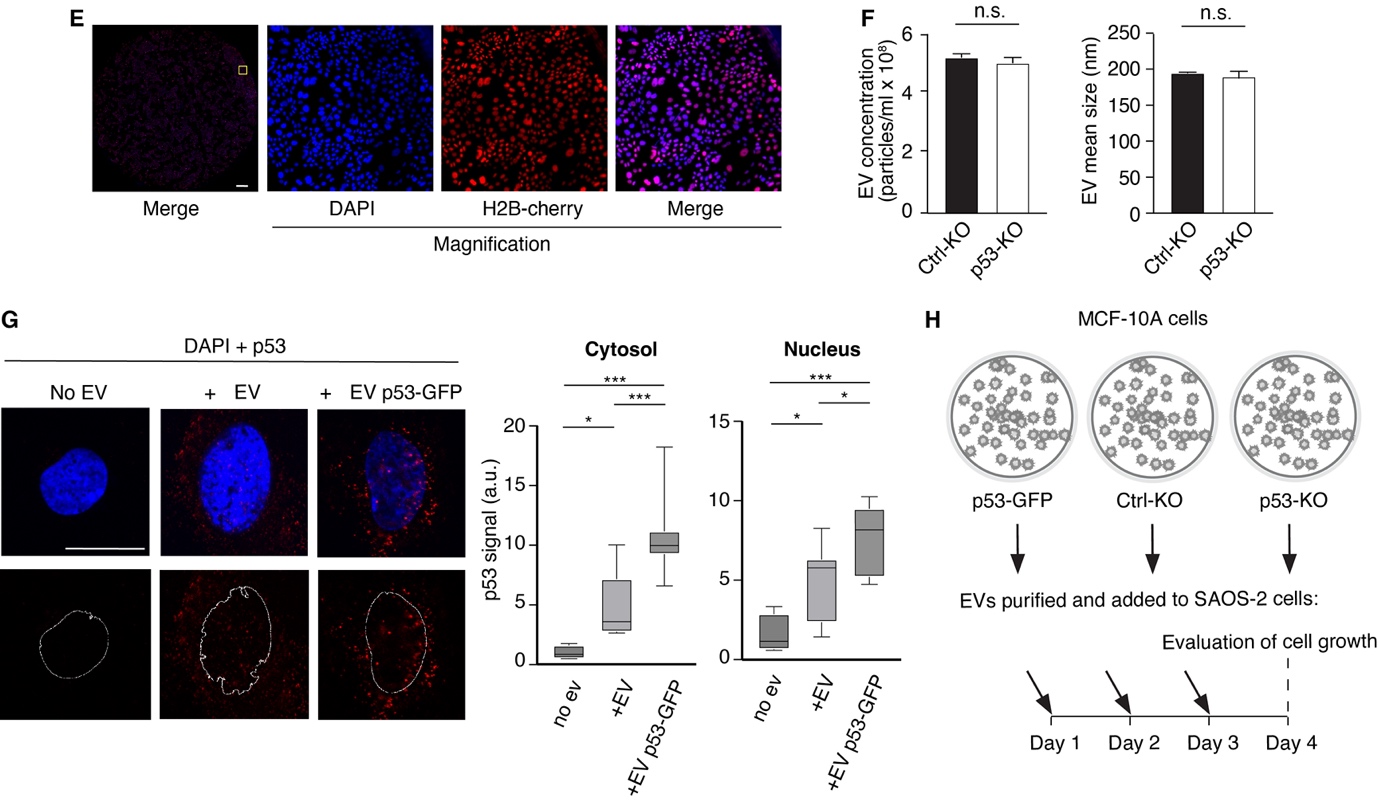
**

**Figure S11. Additional data to Figure 8 of the main text. A.** MCF10A cells Ctrl-KO or p53-KO MCF10A cells were treated with increasing doses of etoposide (as in Figure 7B, C) and analyzed by IB (top) or RT-qPCR (bottom). For the RT-qPCR analysis, data are from three independent experiments and expressed as mean ± SD. ** and ***, p < 0.01 and < 0.001, respectively, *vs*. same condition in p53 KO. Vinculin (VCL) is used as a loading control. **B.** Expi293 cells were transfected as indicated on the right (SNX9-FLAG, NUMB-1-GFP, not-transfected, p53-HA) and EVs were purified from their conditioned medium. EVs were then added to MCF10A-p53-KO recipient cells for 8 h. Recipient cells treated with p53-HA EVs were also treated with etoposide (50 μM) for 8 h. IF of recipient cells was performed as indicated to visualize the tagged constructs (SNX9-FLAG, purple; NUMB-GFP, green; p53-HA, red). Blue, DAPI counterstain. Bar 20 μm. **C**. Cartoon illustrating the MCF10A cell lines expressing LgBiT-Myc-NLS and p53-FLAG-HiBiT, and the reconstitution of NanoLuc through complementation of the HiBiT and LgBiT fragments. **Top:** schematic representation of the HiBiT–LgBiT complementation mechanism, in which the two fragments assemble to form the active NanoLuciferase holoenzyme. The reconstituted enzyme catalyzes the luminescent reaction to generate a detectable light signal. **Bottom:** (i) the donor cell is transfected with a doxycycline-inducible construct encoding the HiBiT fragment fused to p53 (the construct also includes a FLAG tag for detection); (ii) the p53-HiBiT fusion protein is secreted in exosomes; (iii) exosomes are taken up by recipient cells; (iv) p53-HiBiT is released; and (v) subsequently translocates to the nucleus. The recipient cell is engineered to express the LgBiT fragment fused to an NLS (vi), ensuring exclusive nuclear localization (this construct also contains a Myc epitope for detection). Upon reconstitution of the NanoLuc holoenzyme in the nucleus of the recipient cell (vii), luminescence can be detected. **D**. MCF10A cells were stably transfected with the constructs indicated on the right and the expressed proteins were visualized by IF with an anti-Myc antibody (purple, upper panel) or anti-FLAG antibody (purple, bottom panel) and anti-p53 antibody (red). Blue, DAPI counterstain. Bar, 20 μm. Where indicated, cells were also treated with doxycycline for 24 hours. **E**. The coverslips harvested as per point d in Figure 8E were analyzed for possible contamination of cells from the co-culture plate. Coverslips were counterstained with DAPI and analyzed for coincidence of the DAPI and H2B-Cherry signals. The leftmost panel shows an entire coverslip; two coverslips for a total of 100,553 cells were acquired using a HC PL FLUOTAR 10X objective mounted on a DMi8 inverted microscope (Thunder Imaging System - Leica Microsystems). The yellow inset refers to the region of the coverslip magnified in the other three panels. In the two analyzed coverslips, the % of overlap between the DAPI and H2B-Cherry signals was 99.8 and 99.3%. We concluded that virtually no cross-contamination of the coverslips by the co-cultivated cells occurred. Bar 1 mm. **F.** Since p53 has been involved in exosome biogenesis, we purified EVs from MCF10A Ctrl-KO and p53-KO cells and analyzed them by NanoSight NTA for number/concentration (left) and size (right). Results are expressed as means ± SD of three technical replicates. n.s., not significant. Results show that there is no significant difference in EV concentration or size between the two cell populations. **G.** SAOS2 cells were treated for 15 h with EVs purified from the conditioned medium of MCF10A cells (+ EV) or MCF10A-p53-GFP cells (+EV p53-GFP) or left untreated (No EV). Cells were also treated with the proteasome inhibitor MG132 (5 mM) to stabilize p53 and facilitate its detection. IF was performed with anti-p53 (red) and DAPI (blue). Bar, 20 μm. The mean P53 fluorescence intensity of cells from each condition, both in the cytoplasm and in the nucleus, is presented as a box-and-whisker plot on the right, showing the 25th-75th percentiles (box), the median (line), and the minimum and maximum values (whiskers). Sample sizes: No EV, n = 4; +EV, n = 11; +EV p53-GFP, n = 7. * and ***, p<0.05 and < 0.001, respectively. **H**. Scheme of the experiment of Figure 8G. EVs were purified from the conditioned media of MCF10A-Ctrl-KO, MCF10A-p53-KO, and MCF10A-p53-GFP cells, and added to SAOS2 cells for three days. Cell growth was then evaluated on day 4.

**REFERENCES TO SUPPLEMENTARY INFORMATION**

[1] D. Mellacheruvu, Z. Wright, A. L. Couzens, et al., The CRAPome: a contaminant repository for affinity purification-mass spectrometry data, *Nat Methods* **2013**, *10* (8), 730, <https://doi.org/10.1038/nmeth.2557>.

[2] I. N. Colaluca, A. Basile, L. Freiburger, et al., A Numb-Mdm2 fuzzy complex reveals an isoform-specific involvement of Numb in breast cancer, *J Cell Biol* **2018**, *217* (2), 745, <https://doi.org/10.1083/jcb.201709092>.

[3] O. Pylypenko, R. Lundmark, E. Rasmuson, S. R. Carlsson, A. Rak, The PX-BAR membrane-remodeling unit of sorting nexin 9, *EMBO J* **2007**, *26* (22), 4788, <https://doi.org/10.1038/sj.emboj.7601889>.

[4] K. Haberg, R. Lundmark, S. R. Carlsson, SNX18 is an SNX9 paralog that acts as a membrane tubulator in AP-1-positive endosomal trafficking, *J Cell Sci* **2008**, *121* (Pt 9), 1495, <https://doi.org/10.1242/jcs.028530>.

[5] C. Zwahlen, S. C. Li, L. E. Kay, T. Pawson, J. D. Forman-Kay, Multiple modes of peptide recognition by the PTB domain of the cell fate determinant Numb, *EMBO J* **2000**, *19* (7), 1505, <https://doi.org/10.1093/emboj/19.7.1505>.

[6] J. van der Wal, R. Habets, P. Varnai, T. Balla, K. Jalink, Monitoring agonist-induced phospholipase C activation in live cells by fluorescence resonance energy transfer, *J Biol Chem* **2001**, *276* (18), 15337, <https://doi.org/10.1074/jbc.M007194200>.

[7] B. Vu, P. Wovkulich, G. Pizzolato, et al., Discovery of RG7112: A Small-Molecule MDM2 Inhibitor in Clinical Development, *ACS Med Chem Lett* **2013**, *4* (5), 466, <https://doi.org/10.1021/ml4000657>.

[8] K. O'Keefe, H. Li, Y. Zhang, Nucleocytoplasmic shuttling of p53 is essential for MDM2-mediated cytoplasmic degradation but not ubiquitination, *Mol Cell Biol* **2003**, *23* (18), 6396, <https://doi.org/10.1128/MCB.23.18.6396-6405.2003>.

[9] J. M. Stommel, N. D. Marchenko, G. S. Jimenez, et al., A leucine-rich nuclear export signal in the p53 tetramerization domain: regulation of subcellular localization and p53 activity by NES masking, *EMBO J* **1999**, *18* (6), 1660, <https://doi.org/10.1093/emboj/18.6.1660>.

[10] E. Bonsergent, E. Grisard, J. Buchrieser, et al., Quantitative characterization of extracellular vesicle uptake and content delivery within mammalian cells, *Nat Commun* **2021**, *12* (1), 1864, <https://doi.org/10.1038/s41467-021-22126-y>.

[11] A. D. Foers, S. Chatfield, L. F. Dagley, et al., Enrichment of extracellular vesicles from human synovial fluid using size exclusion chromatography, *J Extracell Vesicles* **2018**, *7* (1), 1490145, <https://doi.org/10.1080/20013078.2018.1490145>.

[12] I. N. Colaluca, D. Tosoni, P. Nuciforo, et al., NUMB controls p53 tumour suppressor activity, *Nature* **2008**, *451* (7174), 76, <https://doi.org/10.1038/nature06412>.

[13] N. Louis, C. Evelegh, F. L. Graham, Cloning and sequencing of the cellular-viral junctions from the human adenovirus type 5 transformed 293 cell line, *Virology* **1997**, *233* (2), 423, <https://doi.org/10.1006/viro.1997.8597>.

[14] A. J. Berk, Recent lessons in gene expression, cell cycle control, and cell biology from adenovirus, *Oncogene* **2005**, *24* (52), 7673, <https://doi.org/10.1038/sj.onc.1209040>.

[15] M. Muller, S. Wilder, D. Bannasch, et al., p53 activates the CD95 (APO-1/Fas) gene in response to DNA damage by anticancer drugs, *J Exp Med* **1998**, *188* (11), 2033, <https://doi.org/10.1084/jem.188.11.2033>.

[16] P. A. Link, M. R. Baer, S. R. James, D. A. Jones, A. R. Karpf, p53-inducible ribonucleotide reductase (p53R2/RRM2B) is a DNA hypomethylation-independent decitabine gene target that correlates with clinical response in myelodysplastic syndrome/acute myelogenous leukemia, *Cancer Res* **2008**, *68* (22), 9358, <https://doi.org/10.1158/0008-5472.CAN-08-1860>.
