# Supplementary material for: Endocytic Control of Cell‐Autonomous and Non‐Cell‐Autonomous Functions of p53
Source: Adv Sci (Weinh). 2026 Jan 30;13(30):e13765. doi: 10.1002/advs.202513765 (PMC13248759; doi:10.1002/advs.202513765)
Supplement: Supplementary file 2 — Supporting File 2: advs74070‐sup‐0002‐uncropped_blots.pdf. [file ADVS-13-e13765-s002.pdf]

Fig. 1B

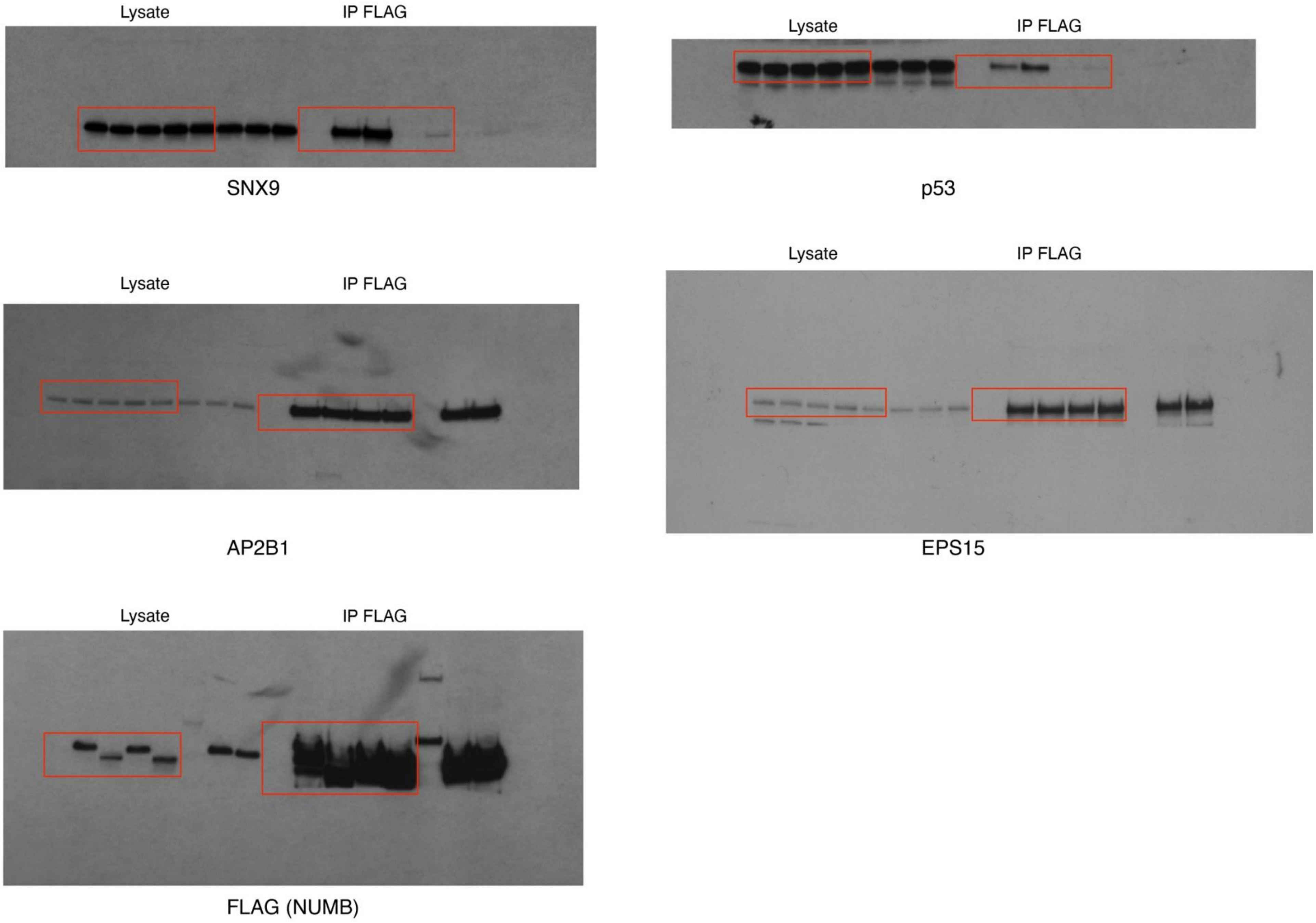

Lysate
IP FLAG
SNX9
Lysate
IP FLAG
p53
Lysate
IP FLAG
AP2B1
Lysate
IP FLAG
EPS15
Lysate
IP FLAG
FLAG (NUMB)

Fig. 1C

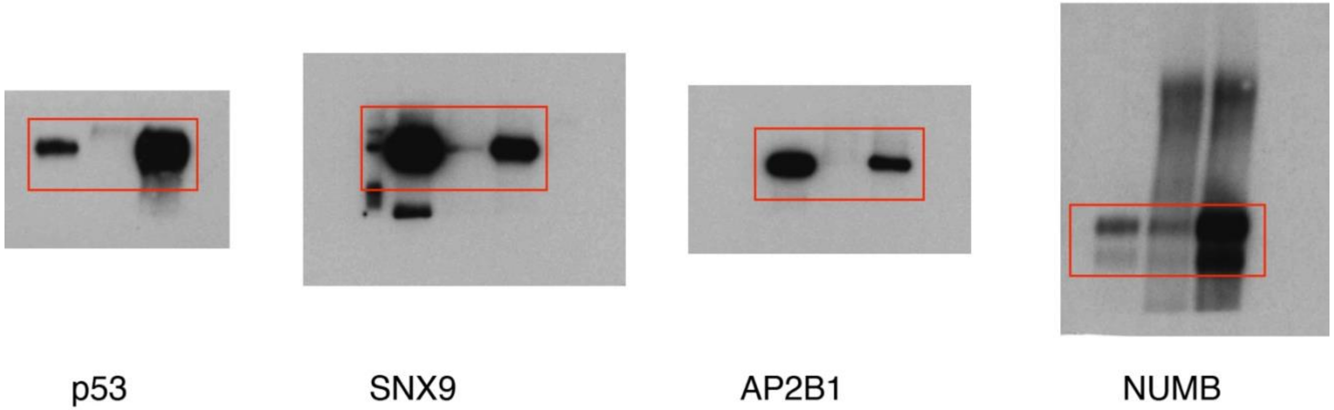


Fig. 1D

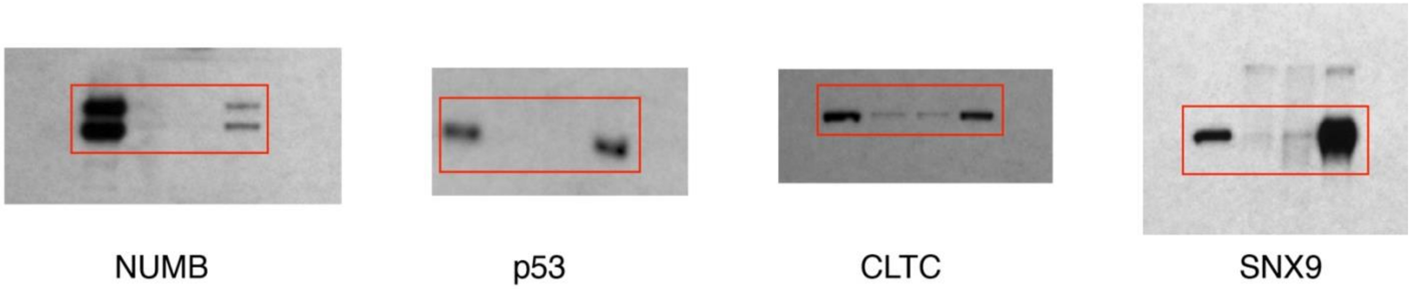

Fig. 1E

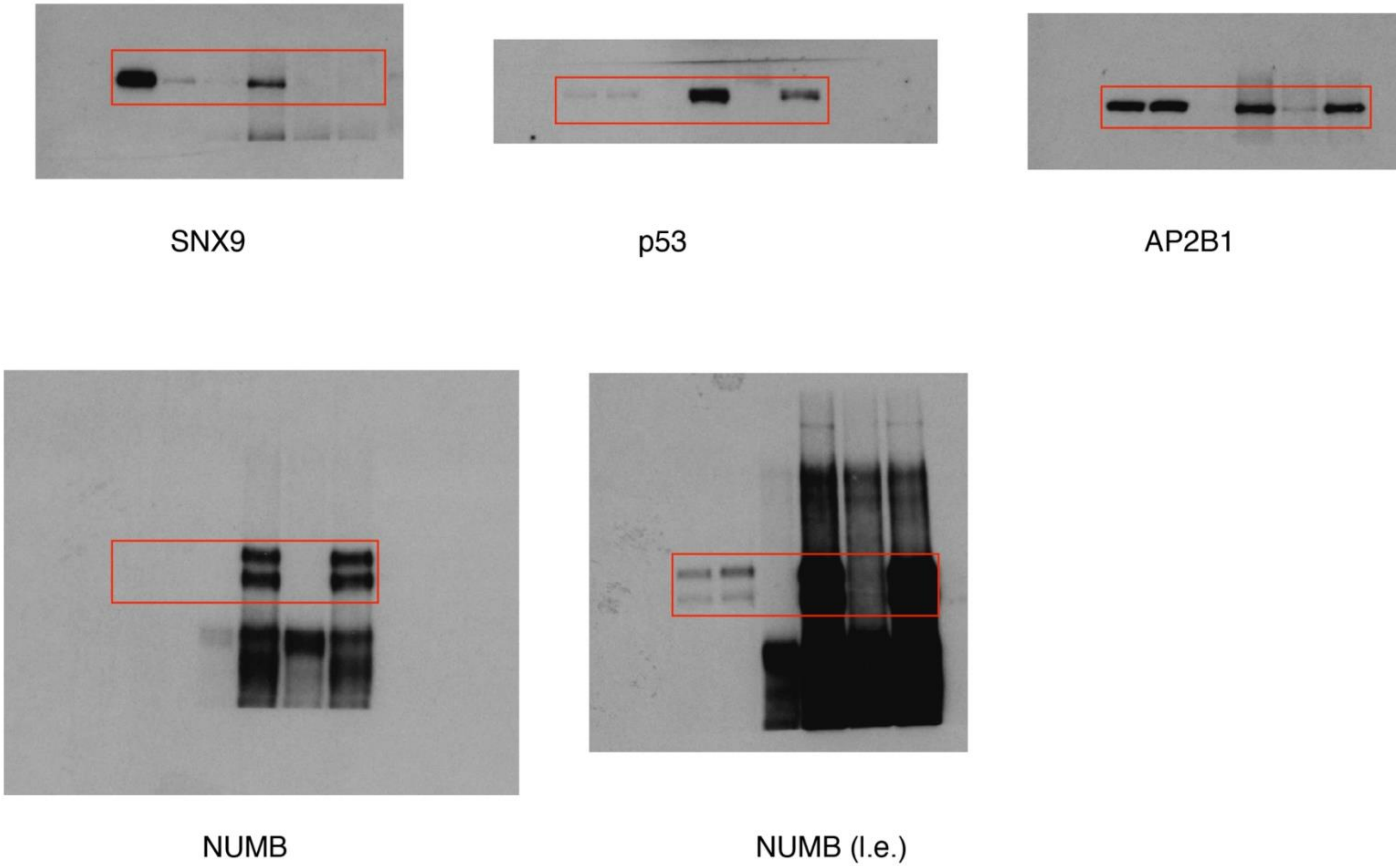

Fig. 1F

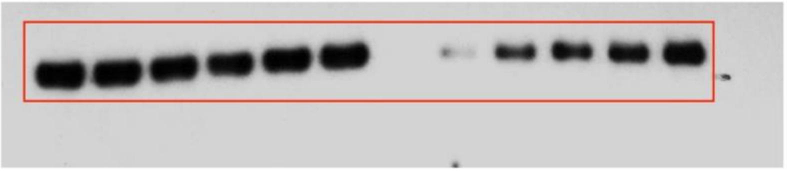

p53

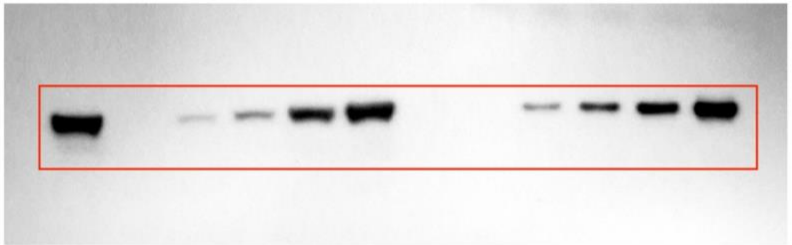

HA (SNX9)

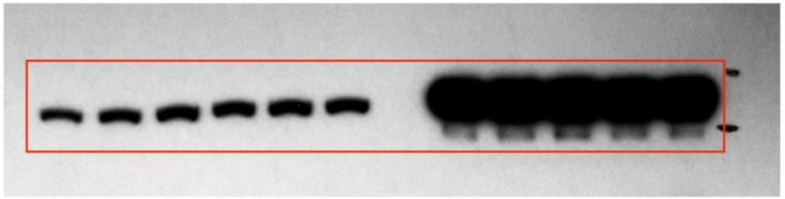

AP2B1 (l.e.)

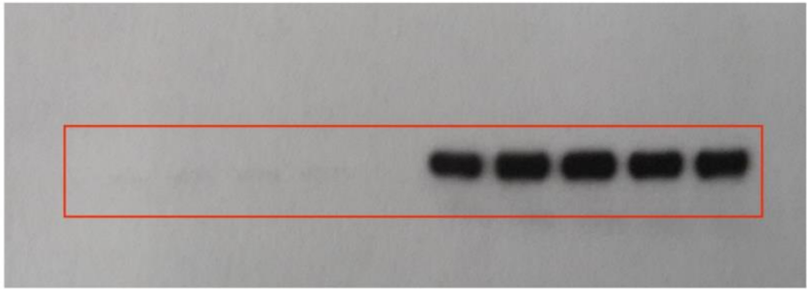

AP2B1

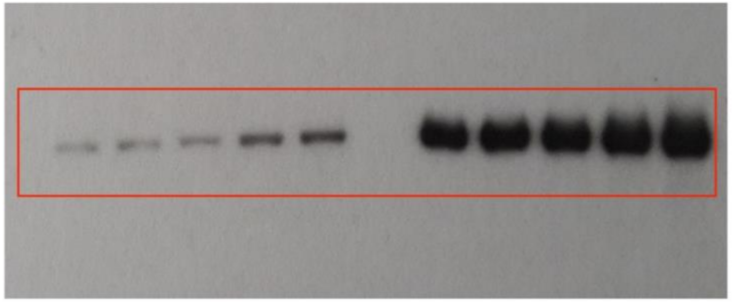

FLAG (NUMB)

Fig. 1G

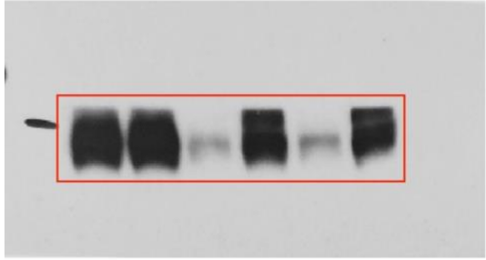

HA (MDM2)

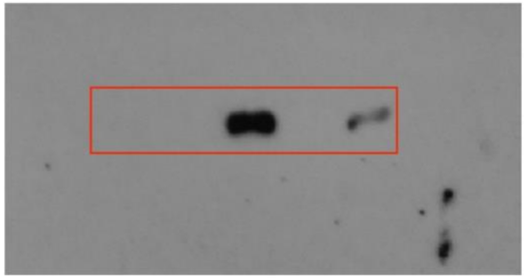

p53

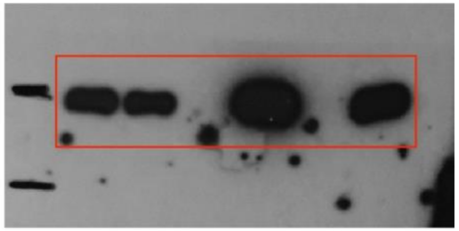

p53 (l.e.)

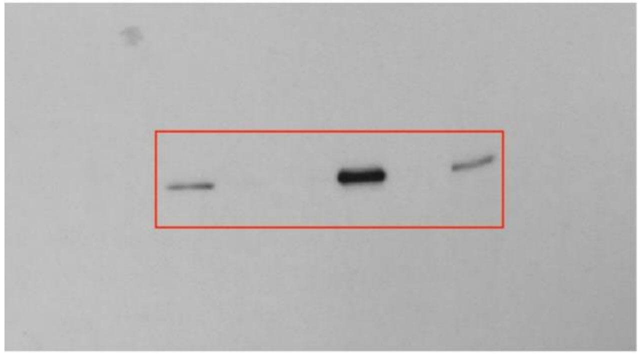

SNX9

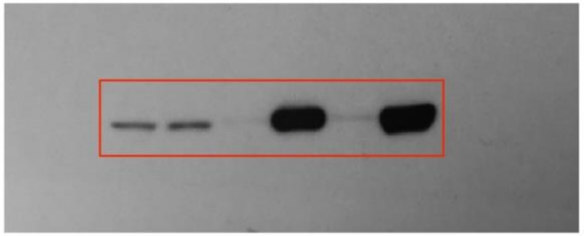

AP2B1

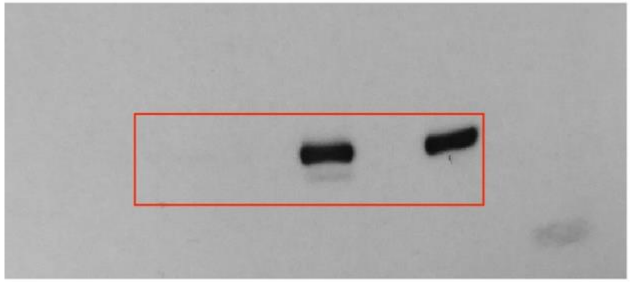

FLAG (NUMB)

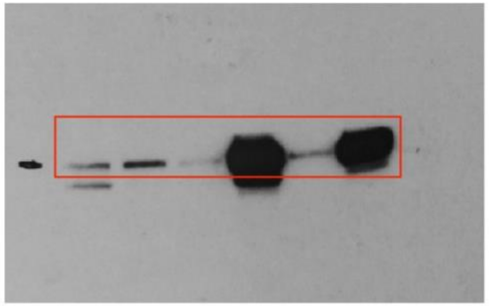

FLAG (NUMB l.e.)

Fig. 1H

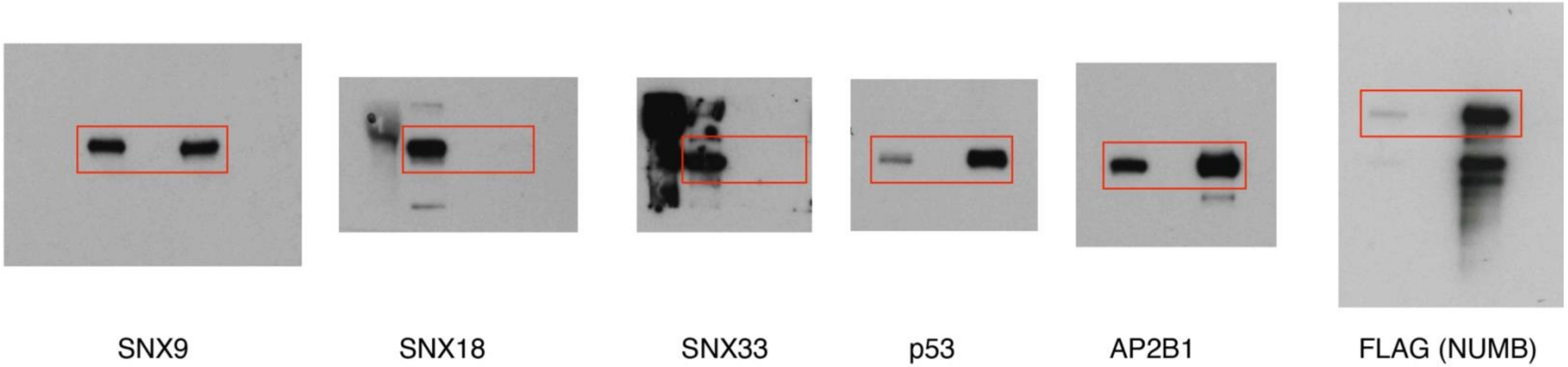


Fig. 2A/ S2A

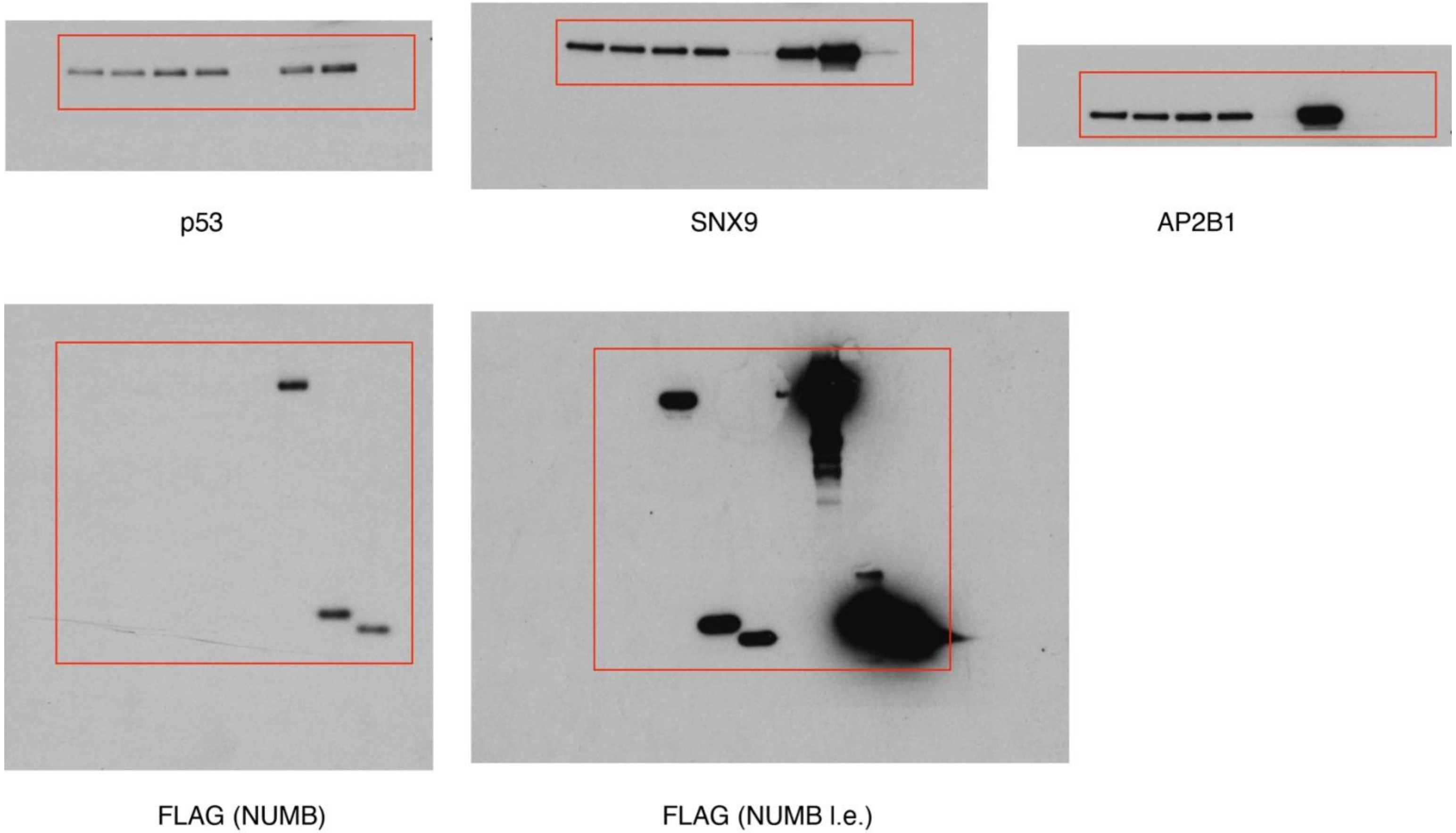

Fig. 2C

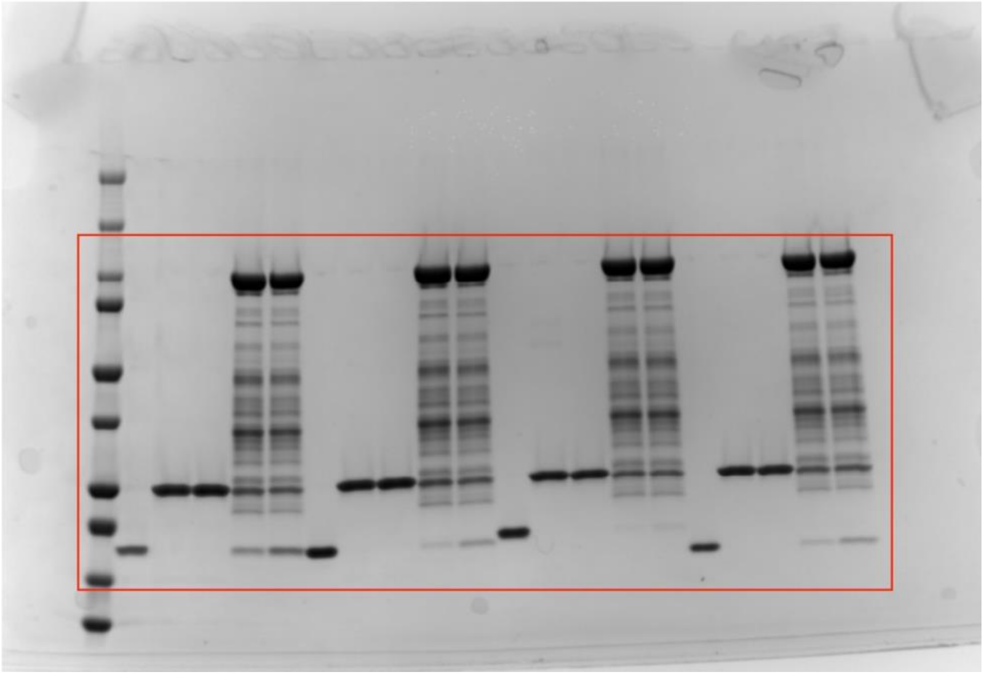

Fig. 2D

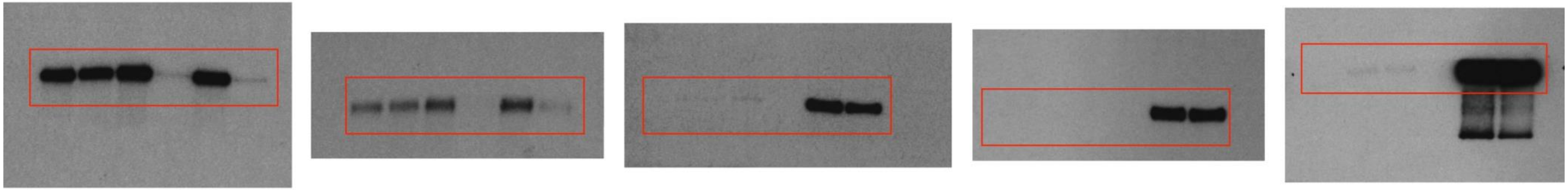

SNX9 p53 AP2B1 FLAG (NUMB) FLAG (NUMB l.e.)

Fig. 3C

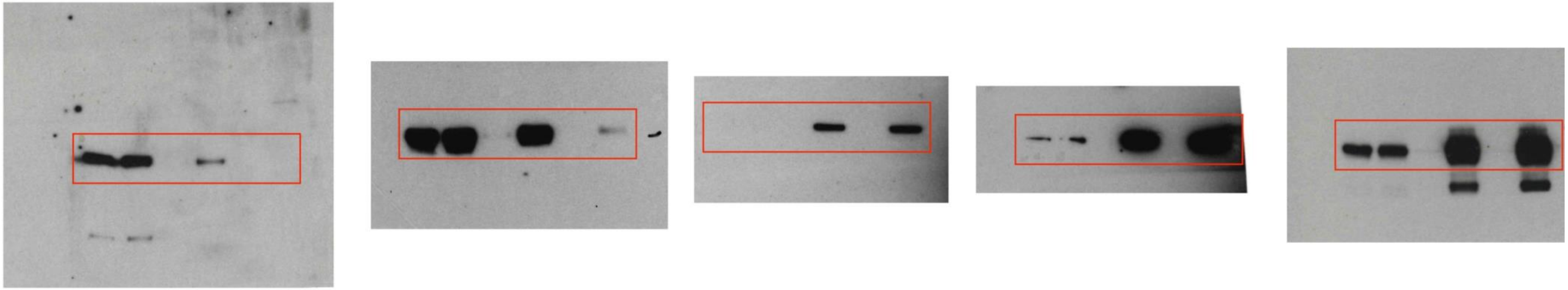

FLAG (NUMB) p53 DNM2 DNM2 (l.e.) HA (SNX9)

Fig. 4A

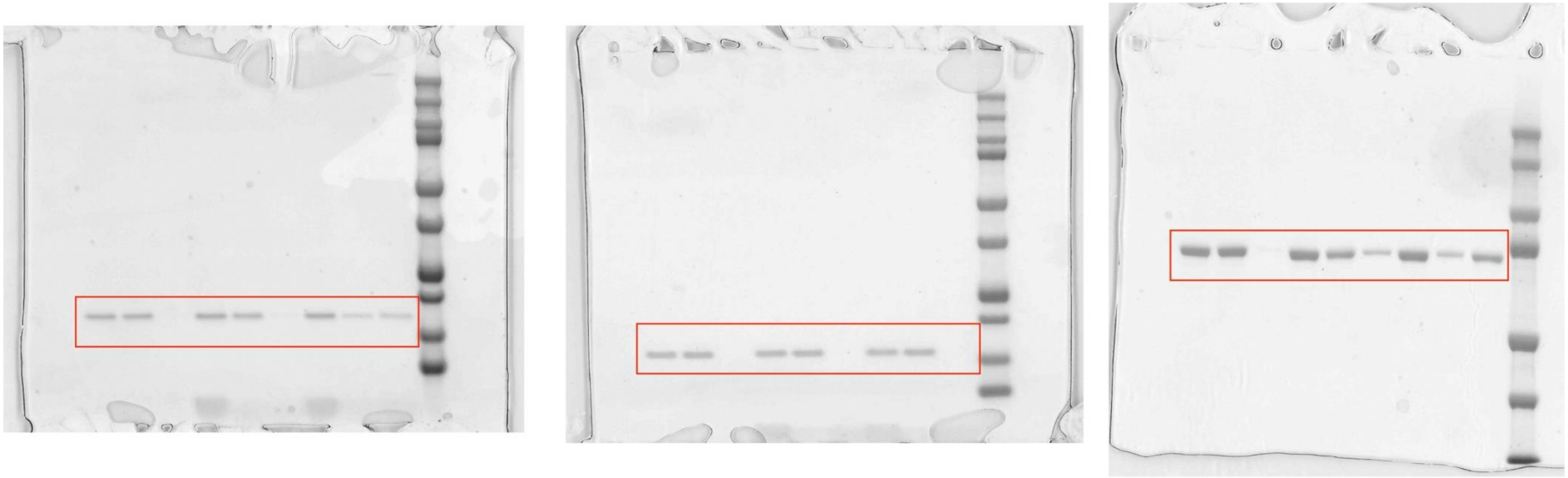

PTB-L PTB-S SNX9-WT

Fig. 4B

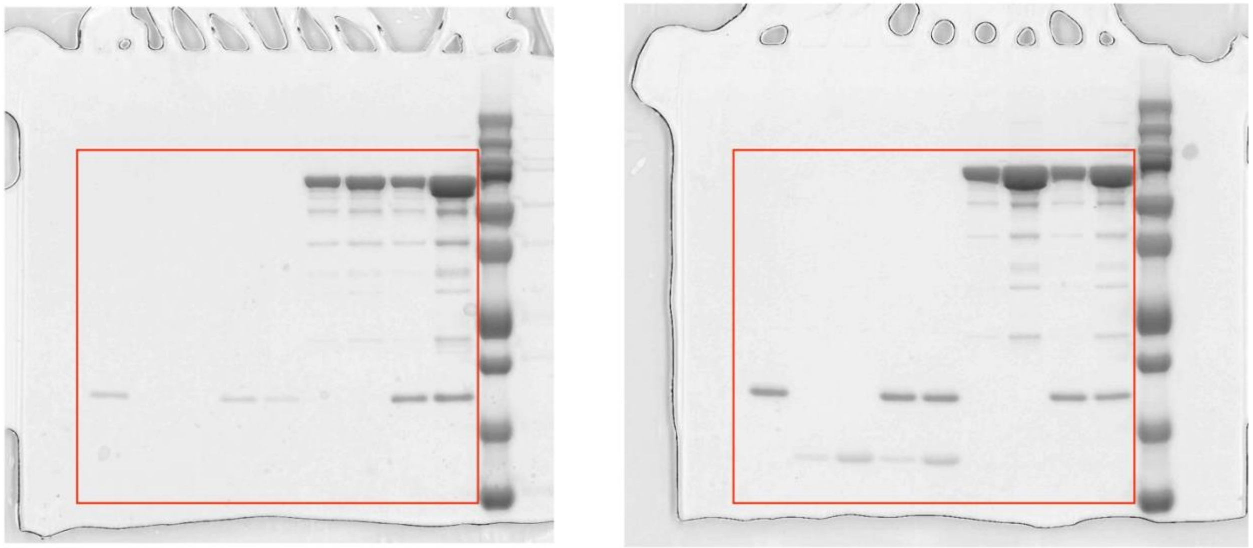

Pellet

Total

Fig. 4C

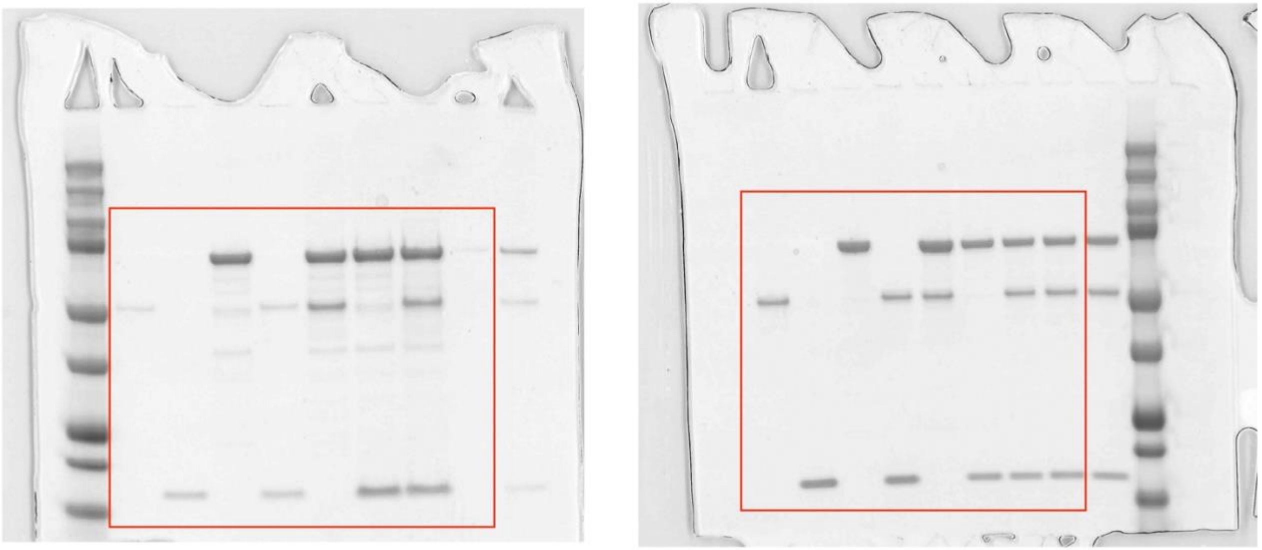

Pellet

Total

Fig. 4D

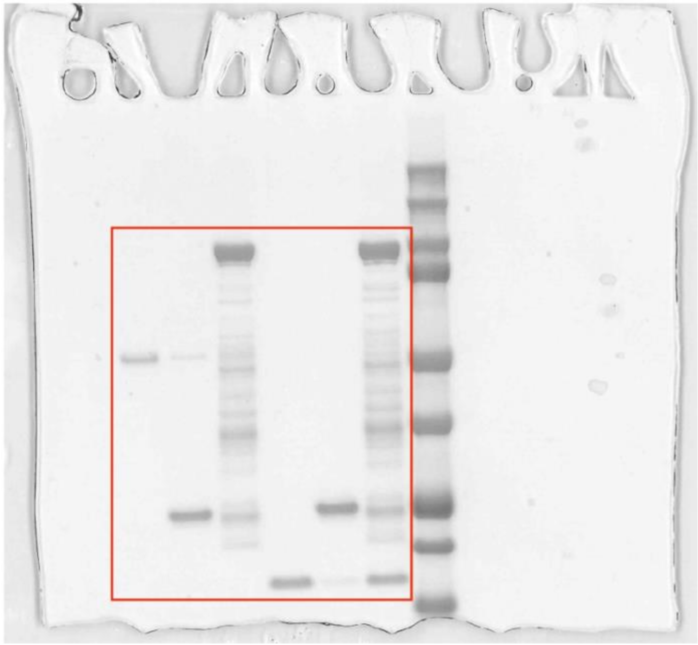

Fig. 6A

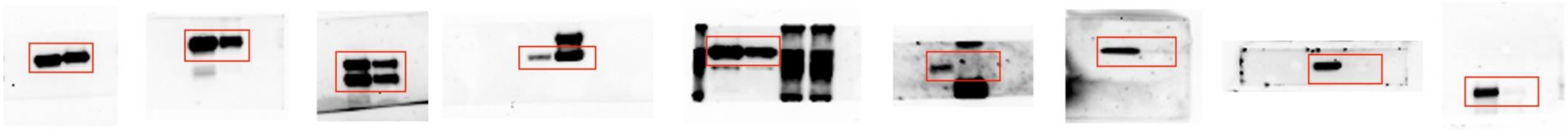

SNX9 p53 NUMB TSG101 FLOT1 BCL2 CYCS HSP90B1 GOLGA2

Fig. 6B

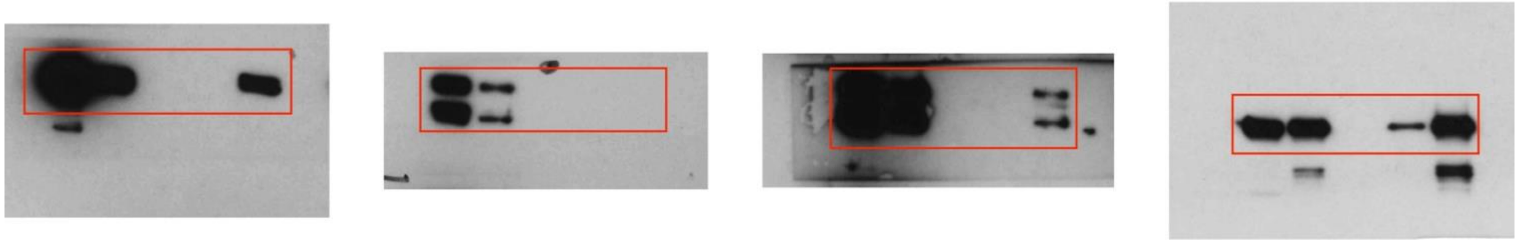

p53 NUMB NUMB l.e. FLAG

Fig. 6C

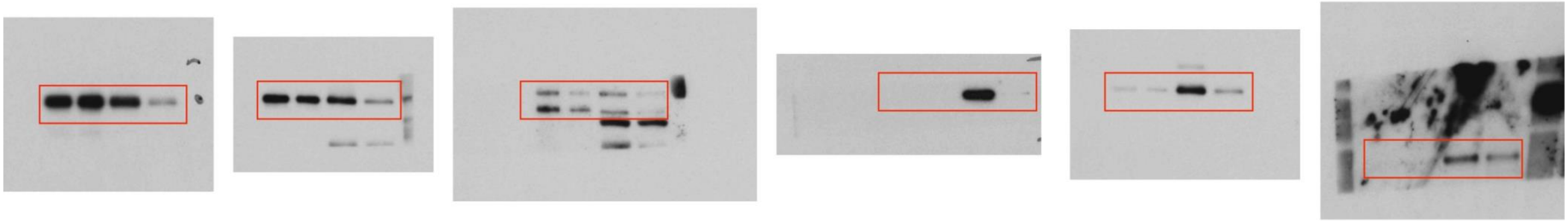

p53 SNX9 NUMB PDCD6IP TSG101 ITGA3

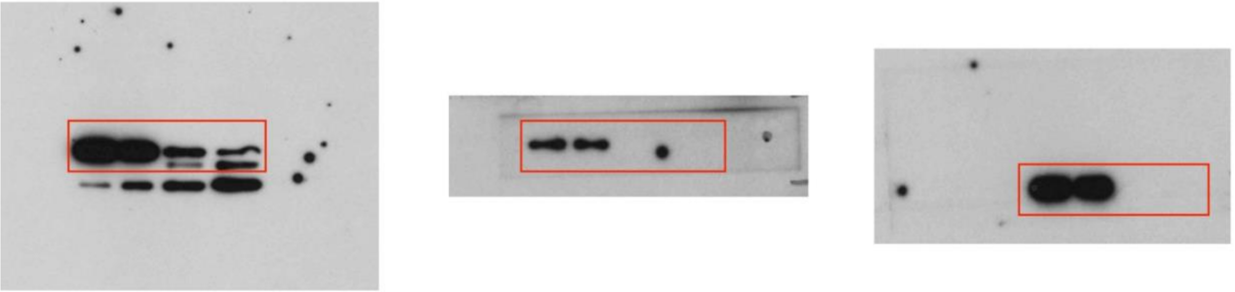

ANXA1 HSP90B1 GOLGA2

Fig. 6F

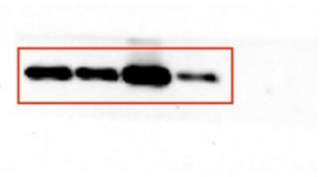

p53

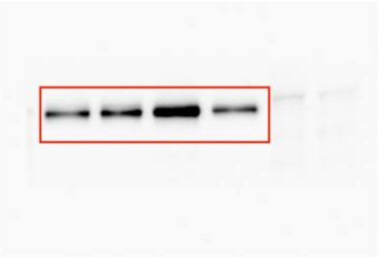

SNX9

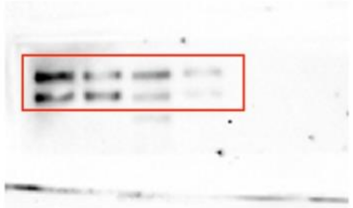

NUMB

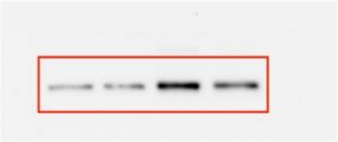

TSG101

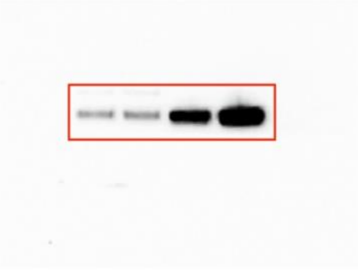

ITGA3

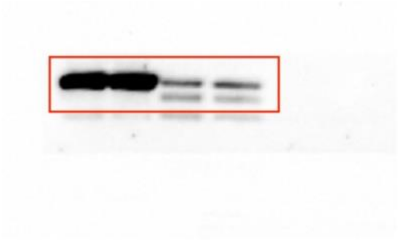

ANXA1

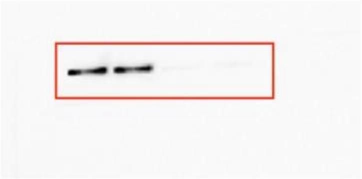

HSP90B1

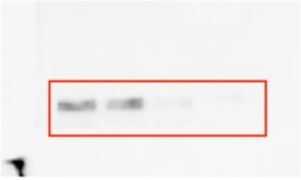

GOLGA2

Fig. 6G

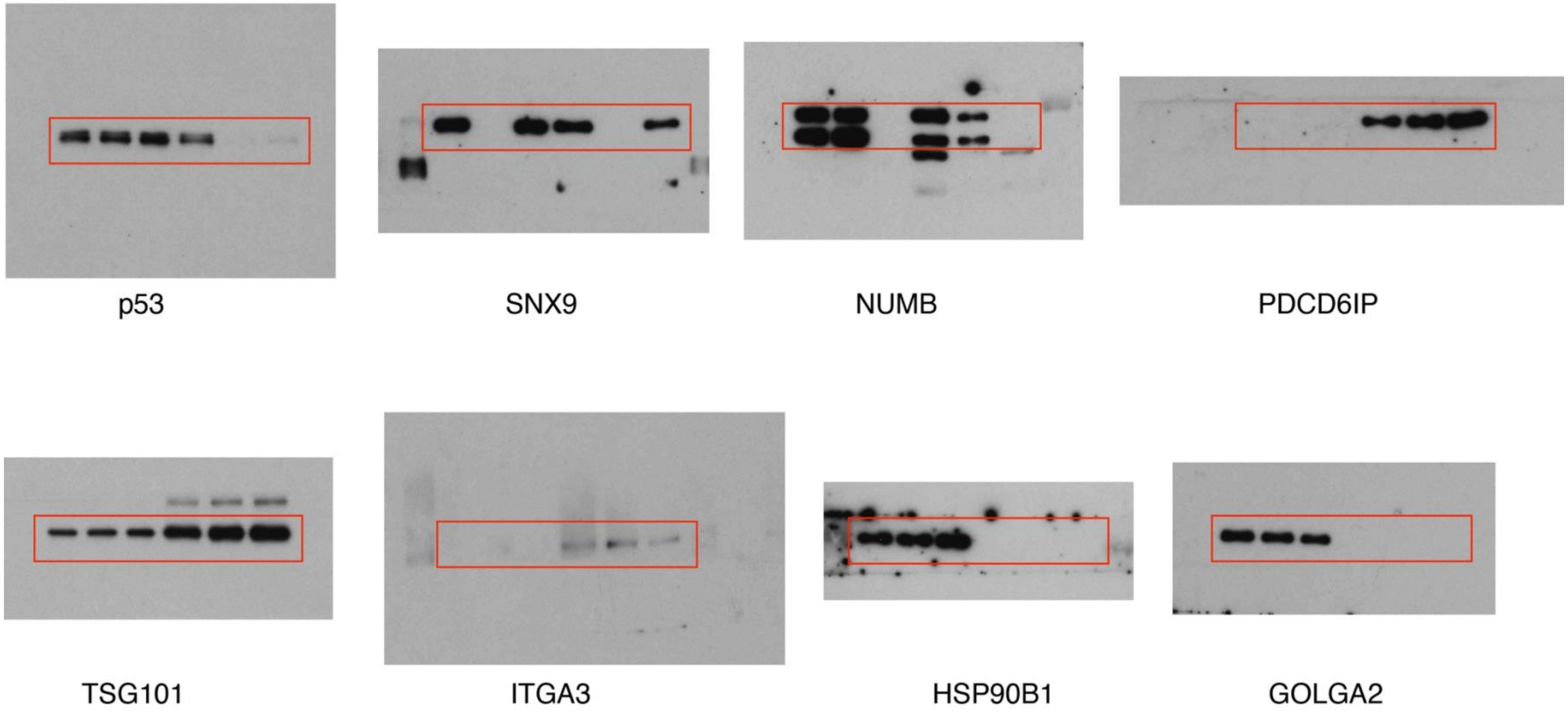

Fig. 7A

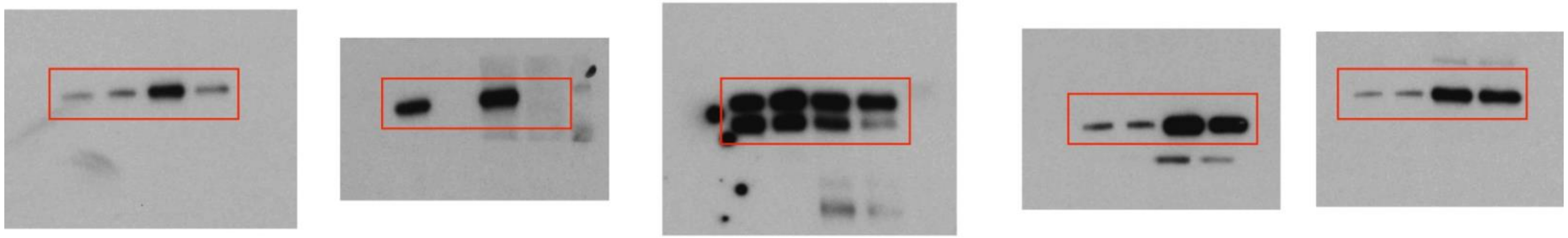

p53 SNX9 NUMB ITGA3 TSG101

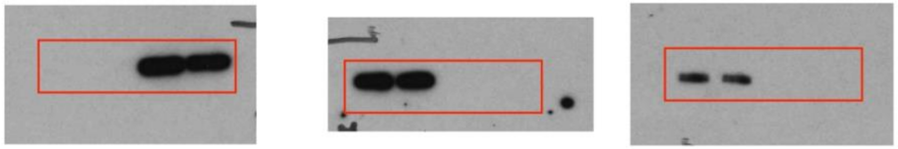

PDCD6IP HSP90B1 GOLGA2

Fig. 7B

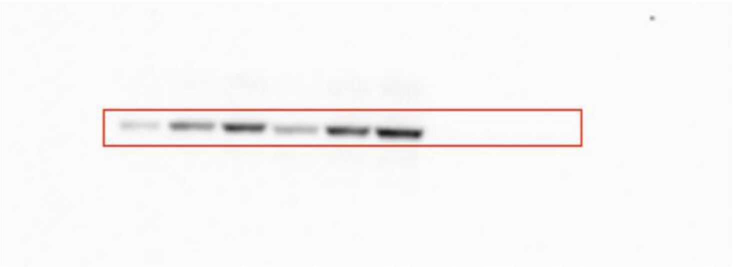

p53

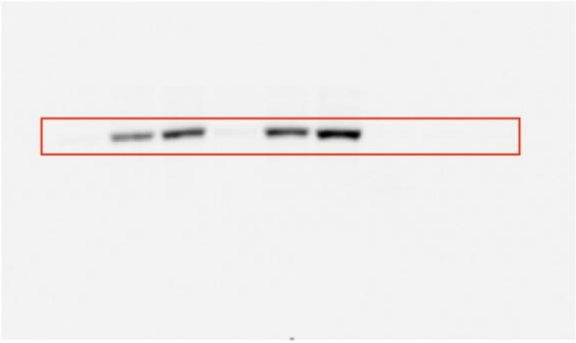

p53$^{ser15}$

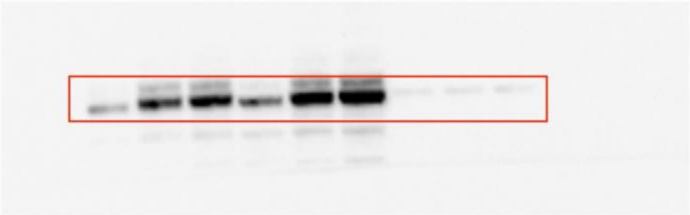

MDM2

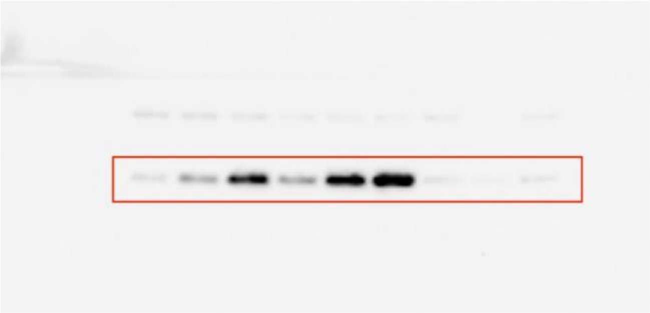

CDKN1A

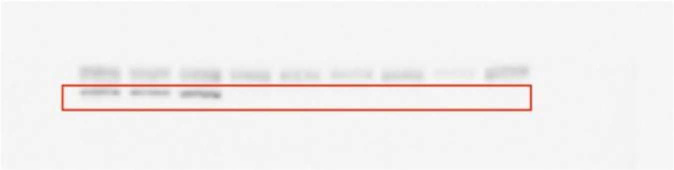

SNX9

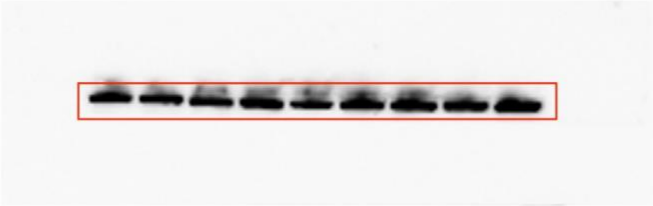

VCL

Fig. S1B

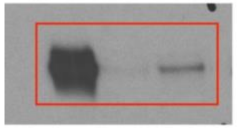

SNX9

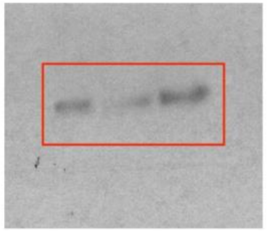

p53

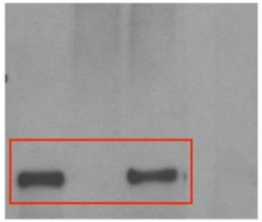

AP2B1

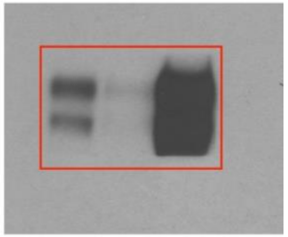

NUMB

Fig. S1C

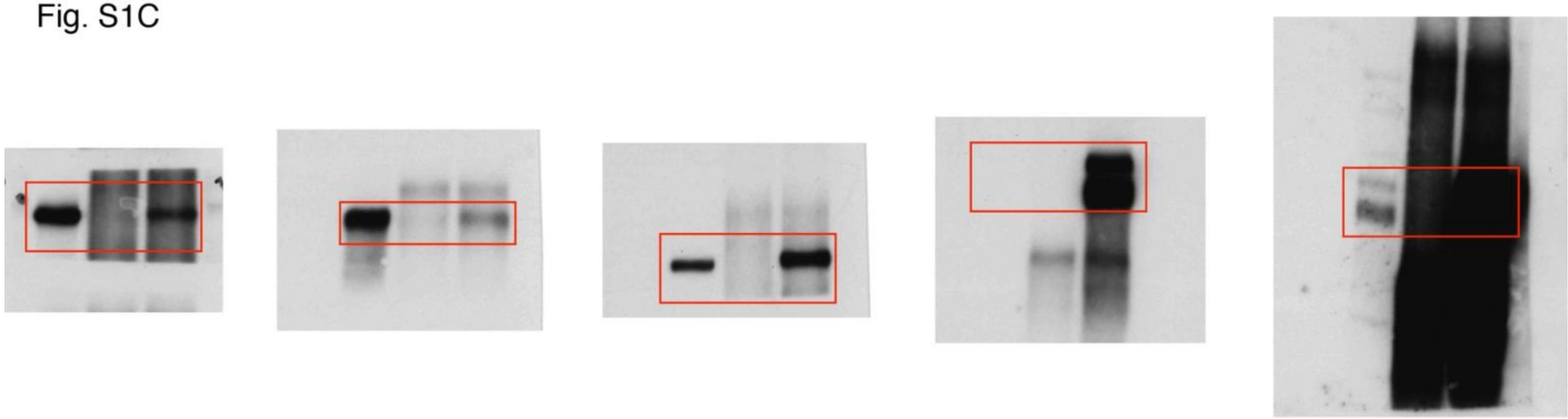

SNX9 p53 AP2B1 NUMB NUMB (l.e.)

Fig. S1D

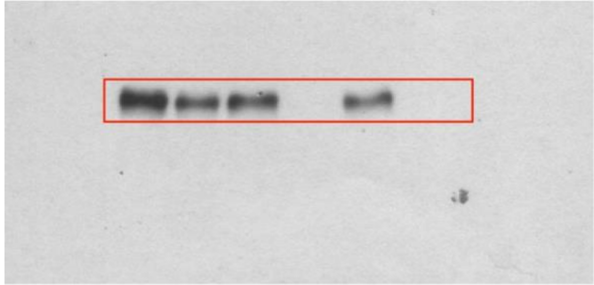

p53

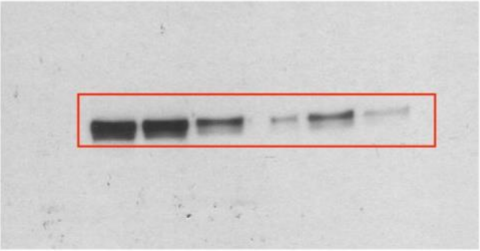

CLTC

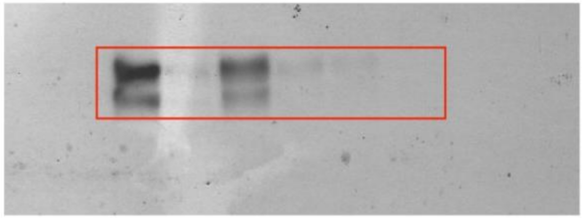

NUMB

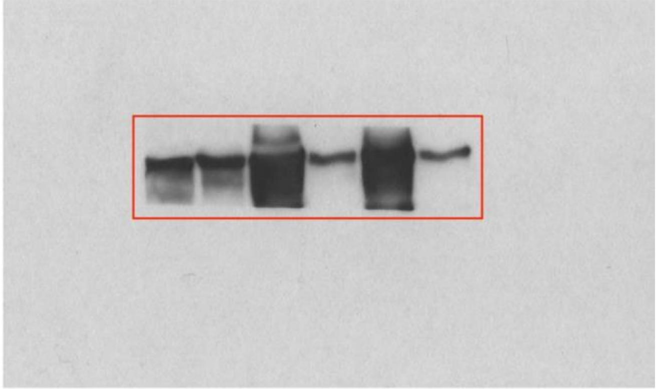

HA (SNX9)

Fig. S1E

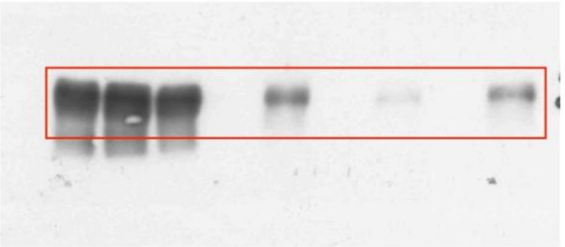

p53

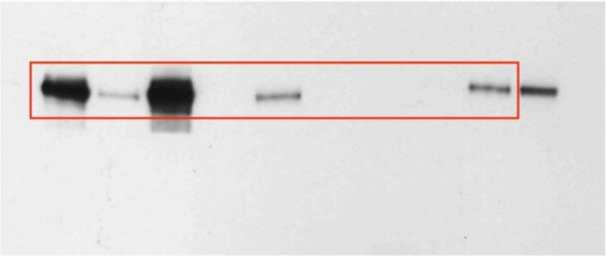

SNX9

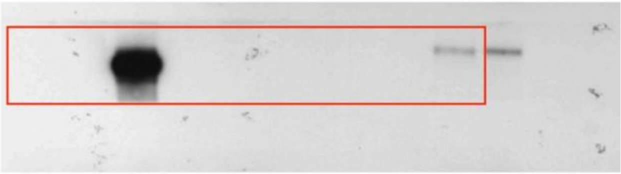

HA (SNX9)

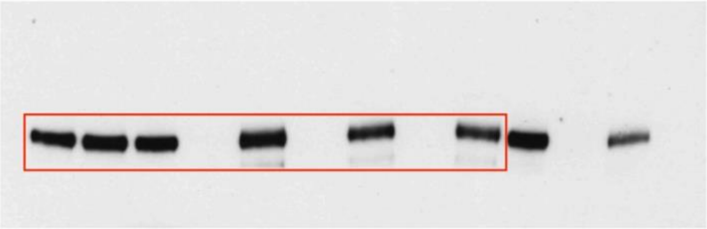

AP2B1

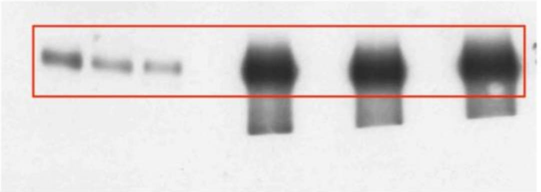

FLAG

Fig. S2B

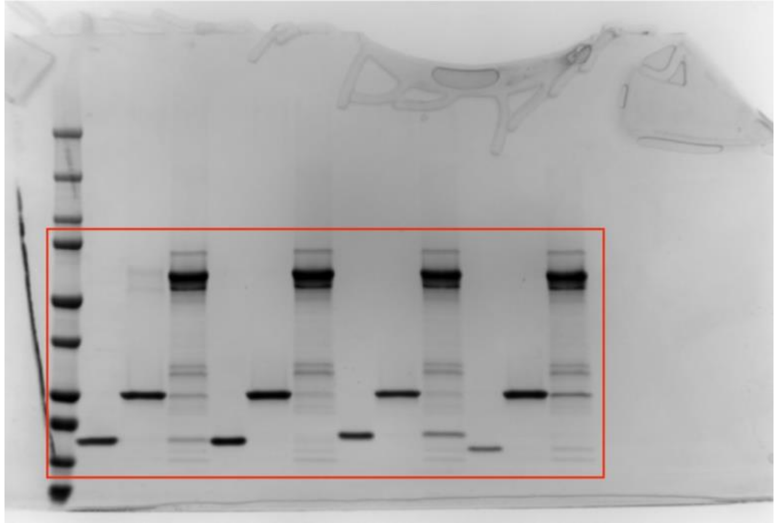

Fig. S3A

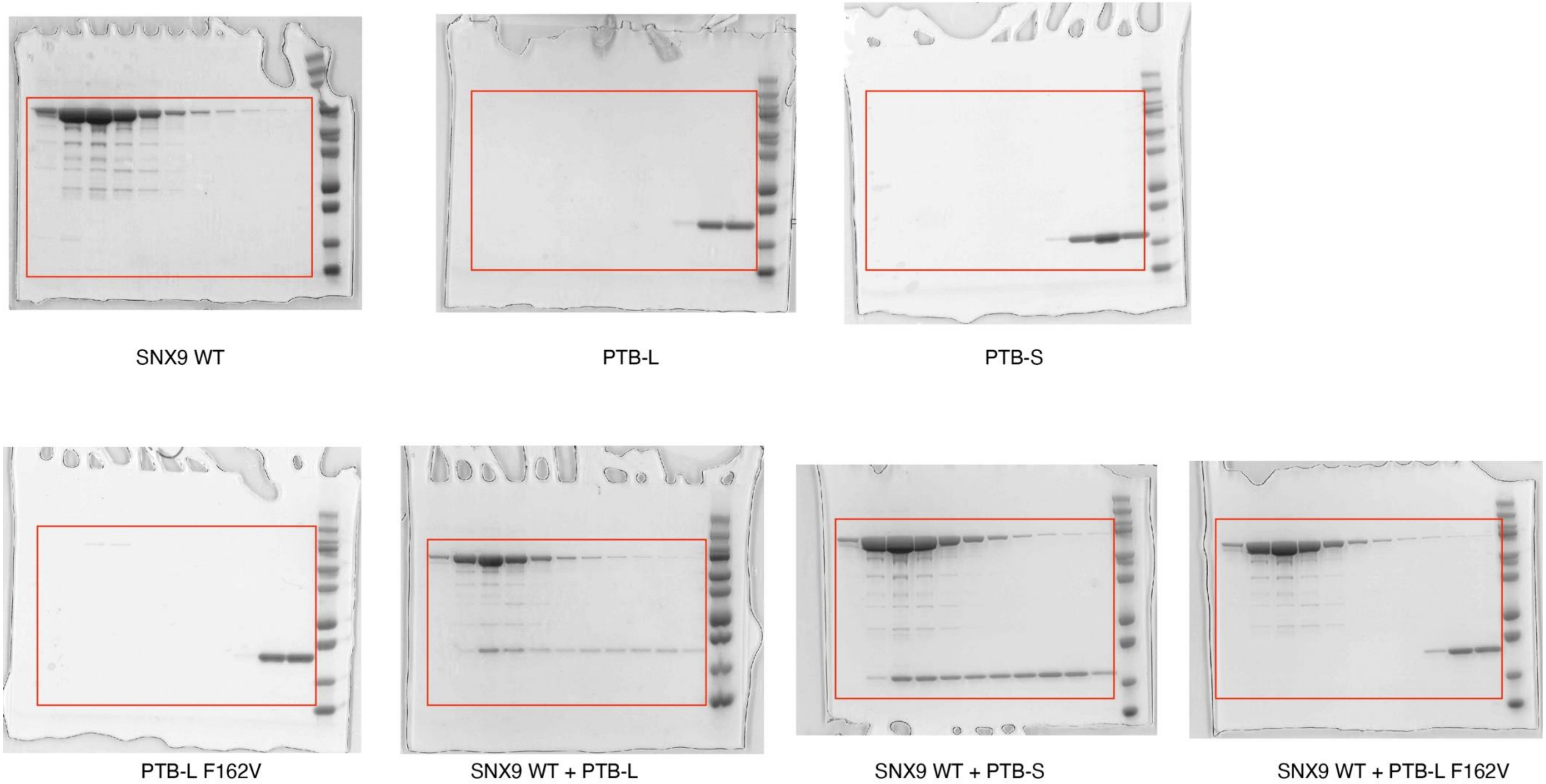
SNX9 WT
PTB-L
PTB-S
PTB-L F162V
SNX9 WT + PTB-L
SNX9 WT + PTB-S
SNX9 WT + PTB-L F162V

Fig. S3C

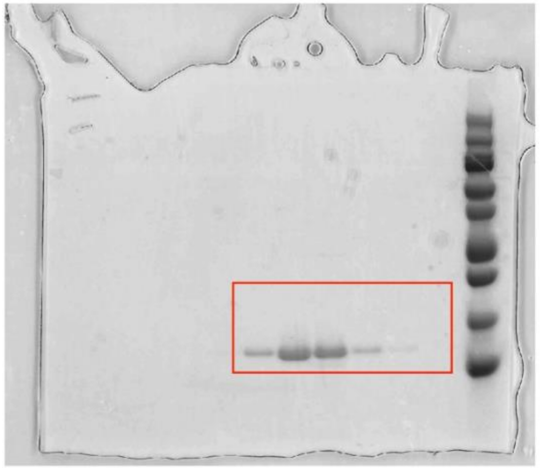

MDM2

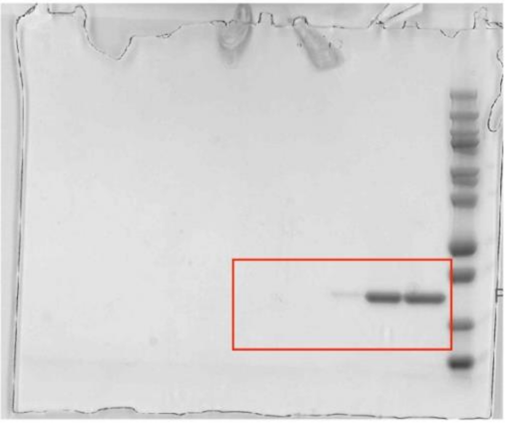

PTB-L

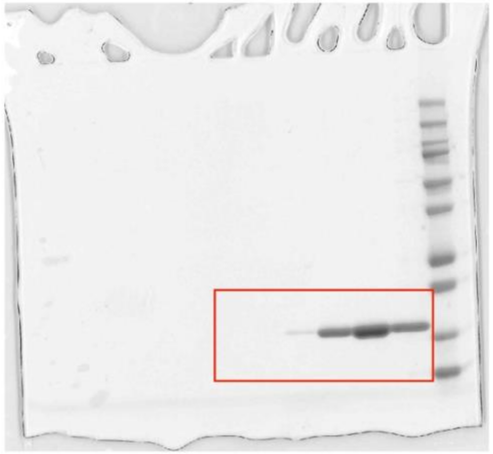

PTB-S

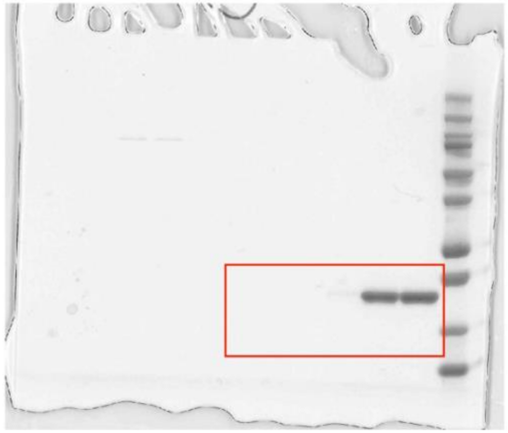

PTB-L F162V

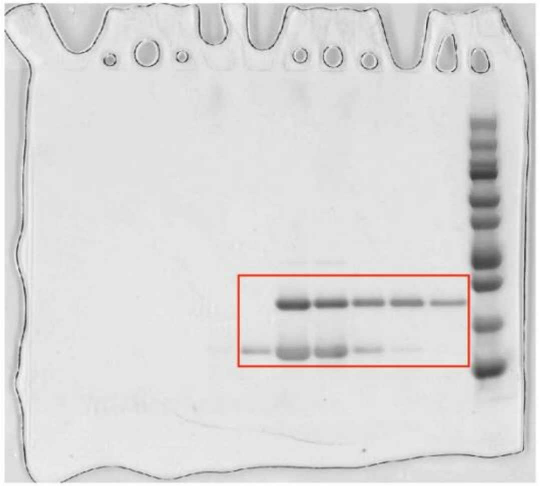

PTB-L + MDM2

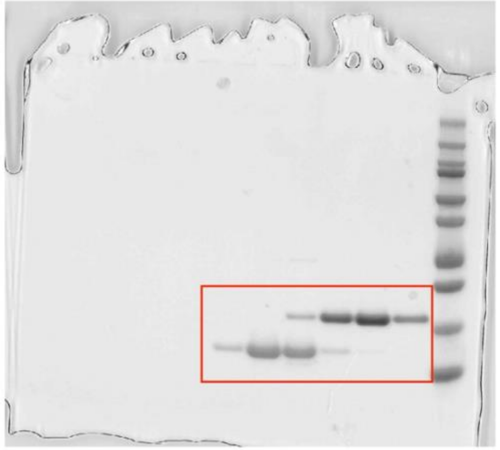

PTB-S + MDM2

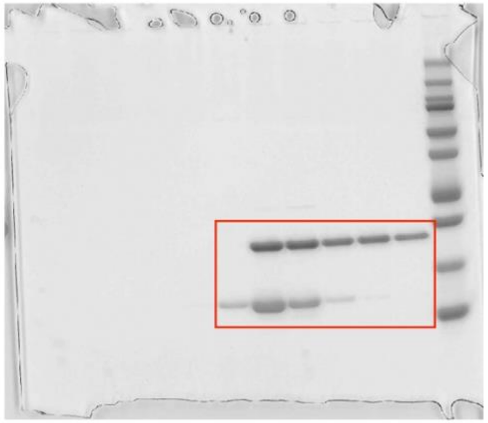

PTB-L F162V + MDM2

Fig. S4A

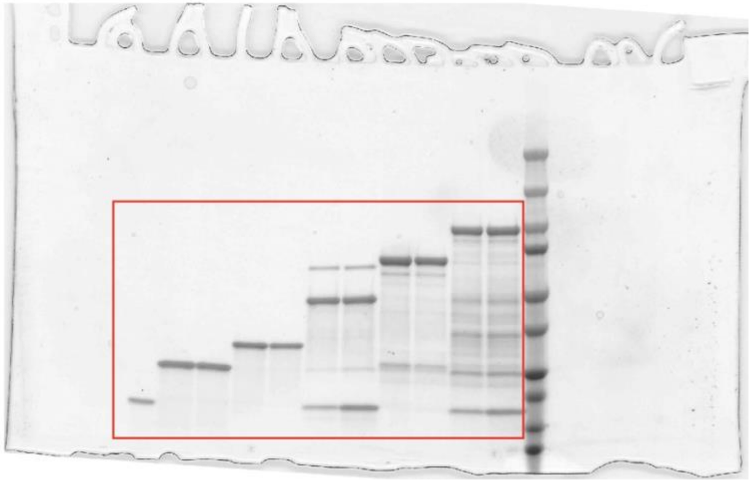

Fig. S4B

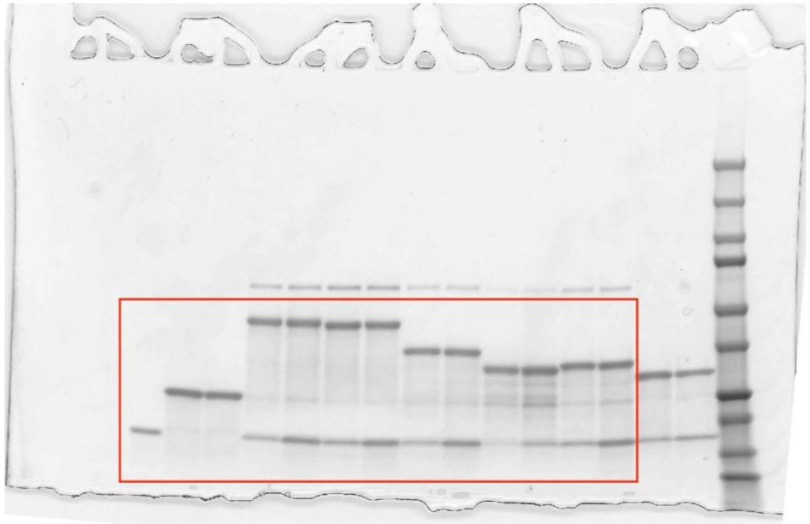

Fig. S4C

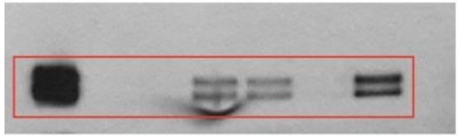
NUMB

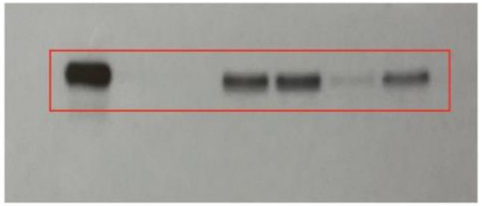
p53

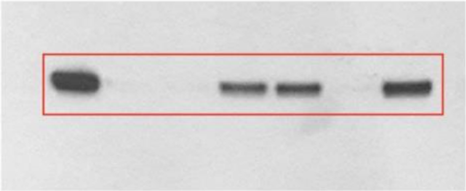
CLTC

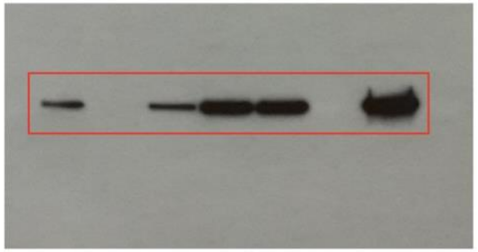
DNM2

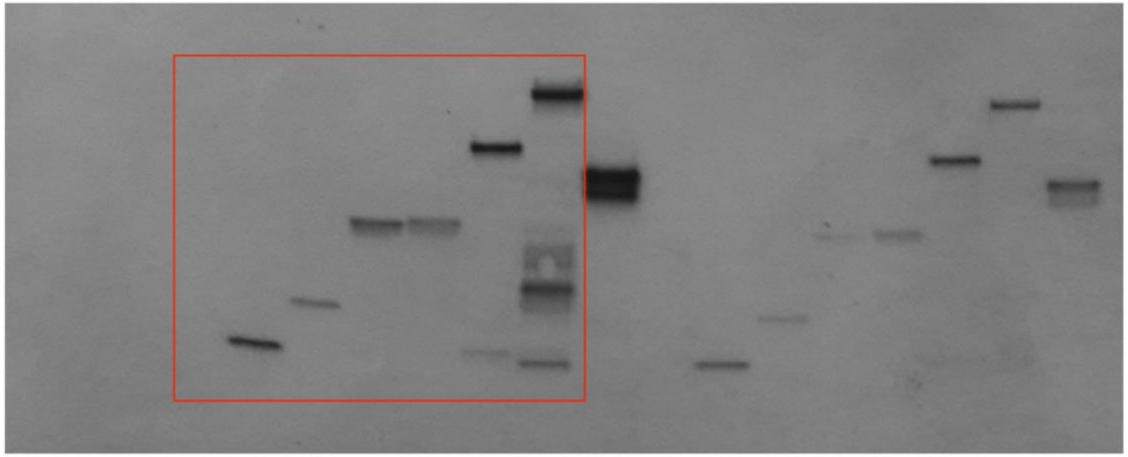
GST

Fig. S4D

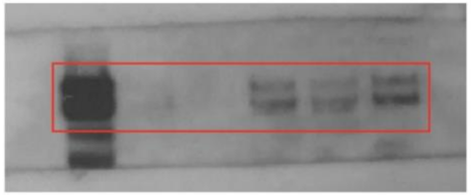

NUMB

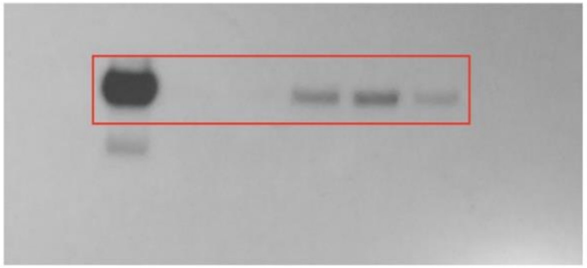

p53

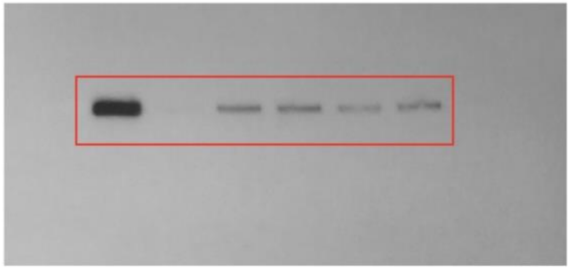

CLTC

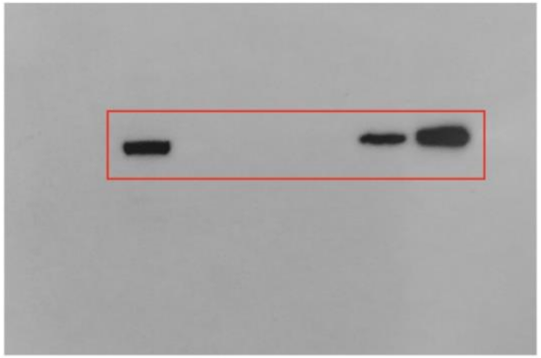

DNM2

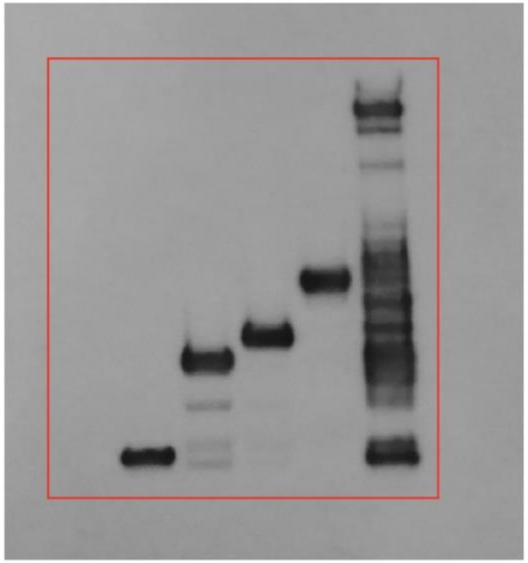

GST

Fig. S4E

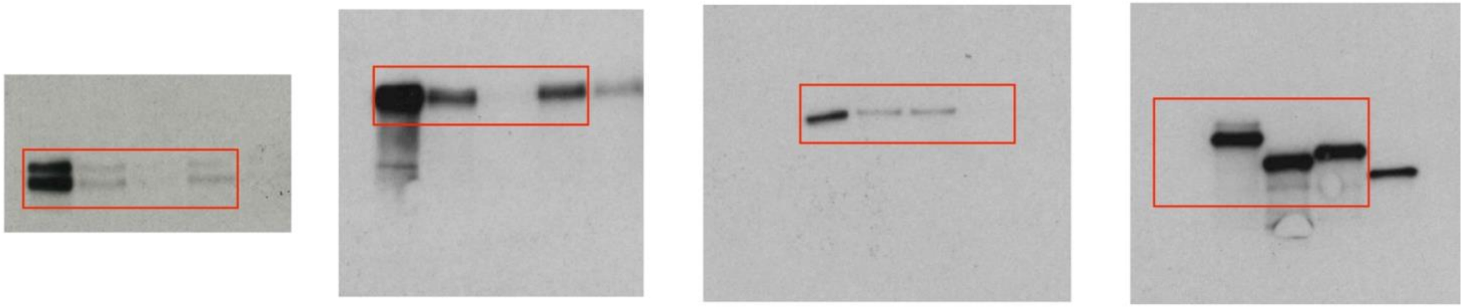

NUMB p53 CLTC GST

Fig. S5C

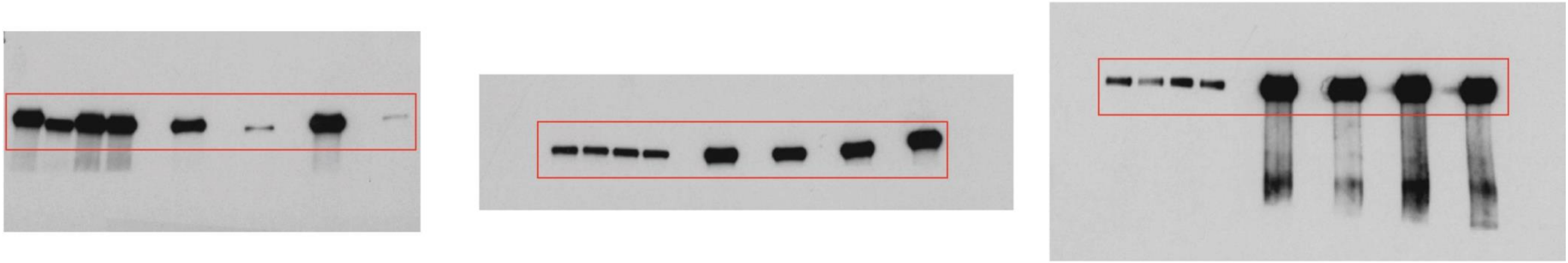

HA (SNX9) AP2B1 FLAG (NUMB)

Fig. S5D

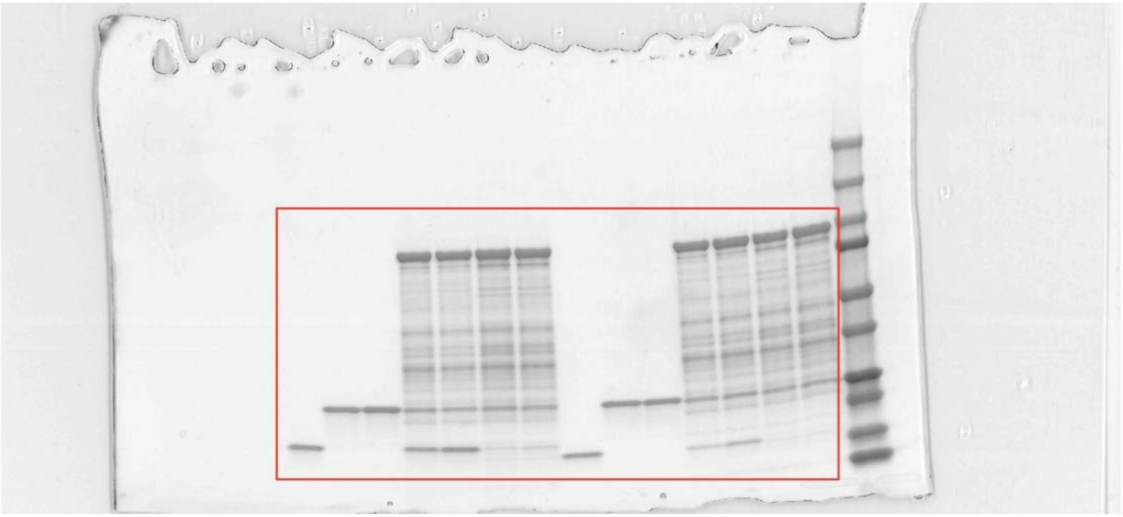

Fig. S6C

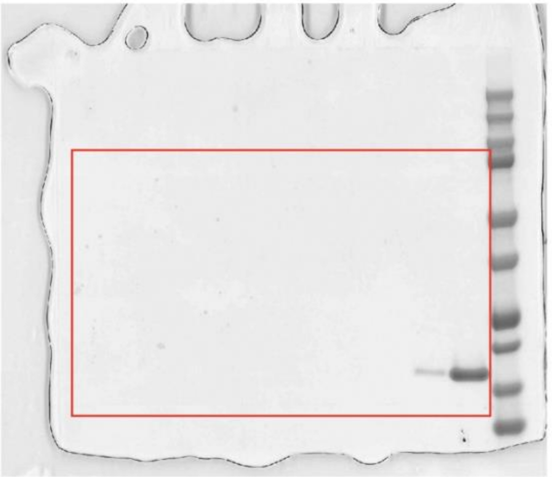

PTB-L

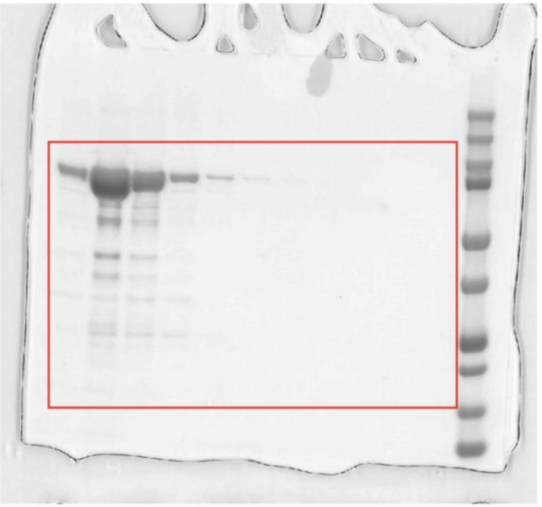

SNX9 WT

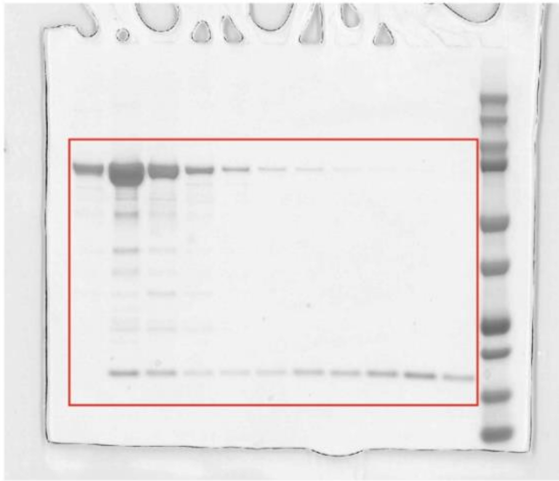

SNX9 WT + PTB-L

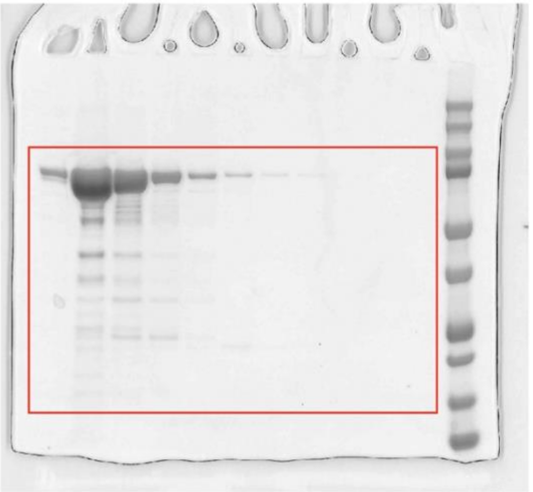

SNX9 PX mut

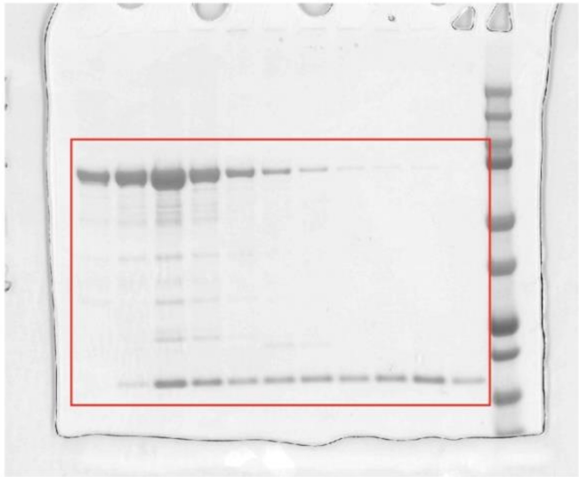

SNX9 PX mut + PTB-L

Fig. S6D

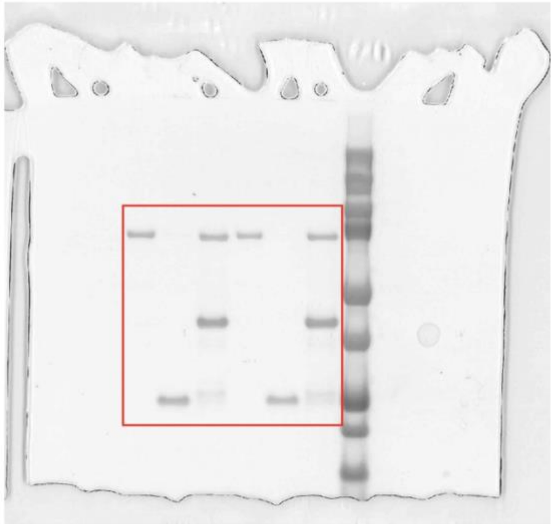

Fig. S7E

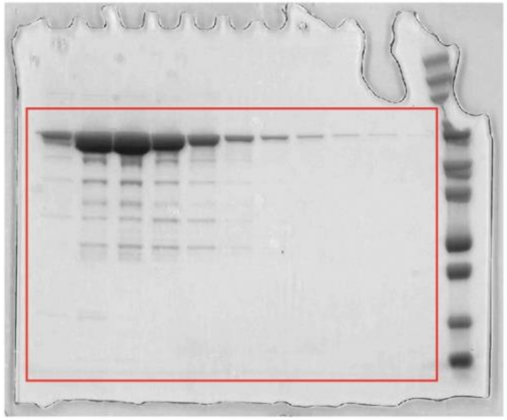

SNX9 WT

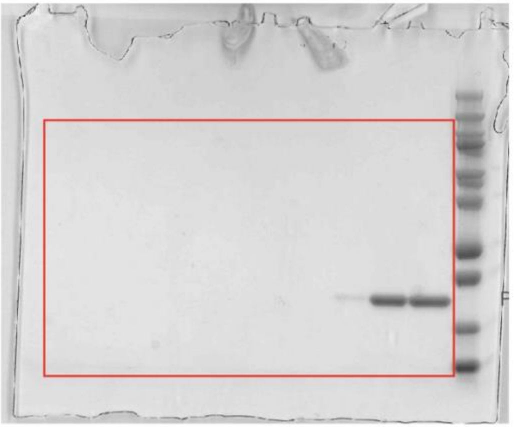

NUMB PTB-L

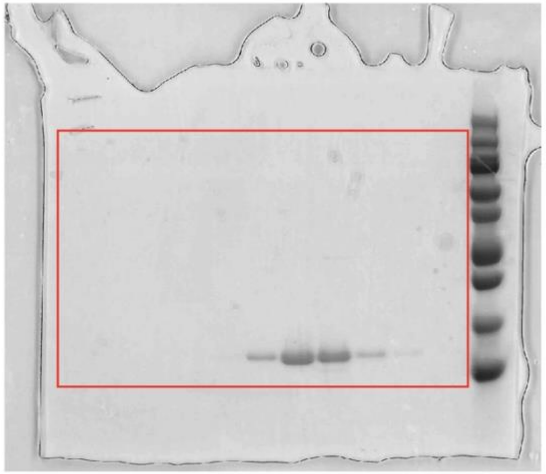

MDM2

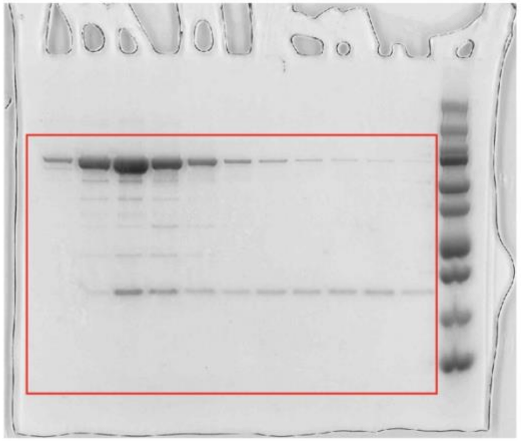

SNX9 WT + NUMB PTB-L

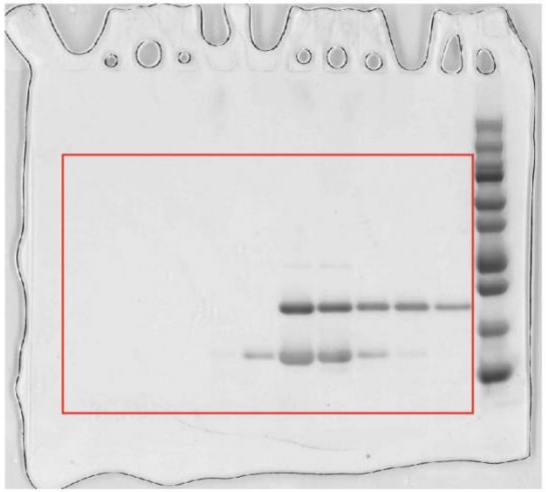

NUMB PTB-L + MDM2

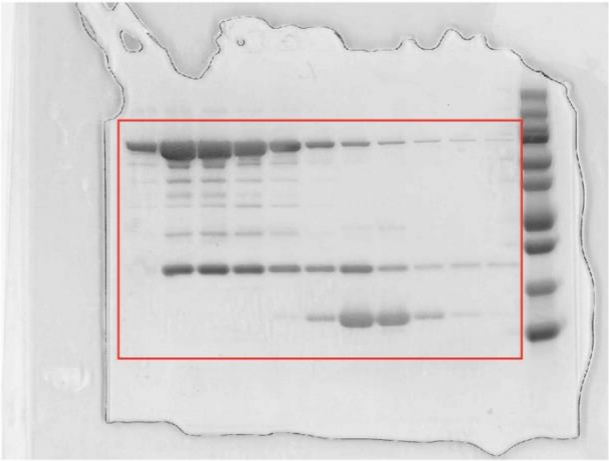

SNX9 WT + NUMB PTB-L + MDM2

Fig. S7F

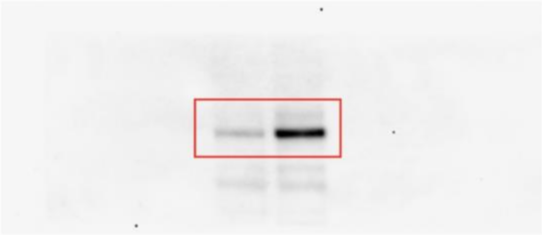

MDM2

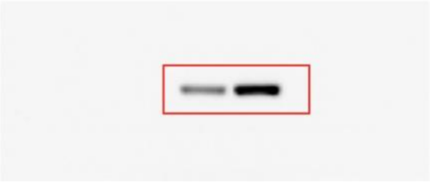

p53

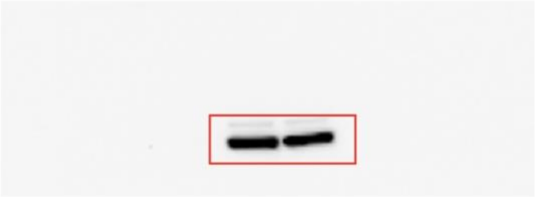

VCL

Fig. S8C

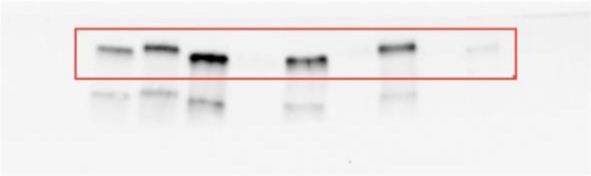

HA(p53)

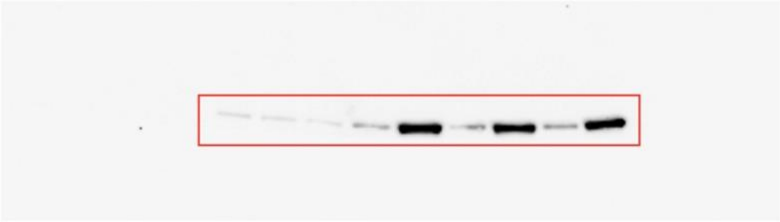

DNM2

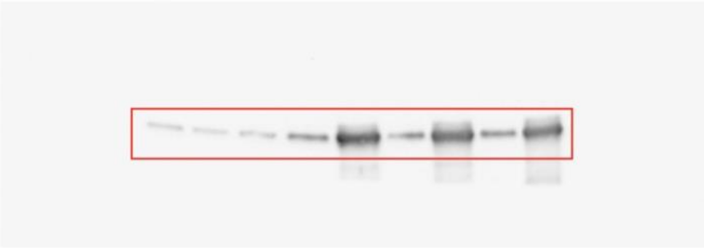

FLAG (SNX9)

Fig. S9B

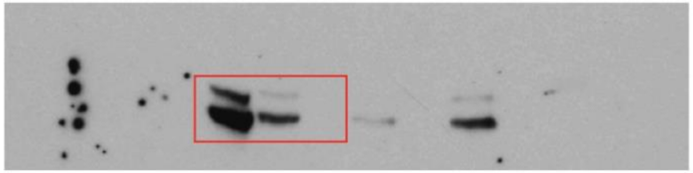
NUMB

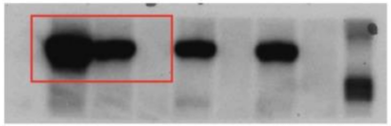
SNX9

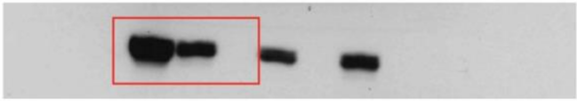
p53

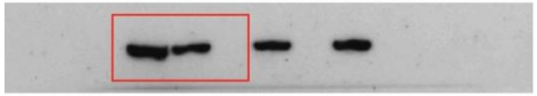
PDCD6IP

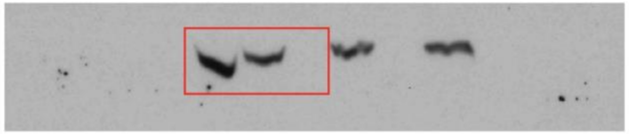
TSG101

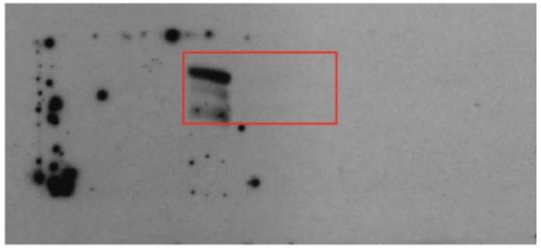
LRP6

Fig. S9C

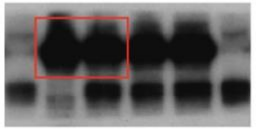
SNX9

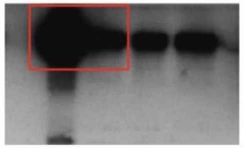
p53

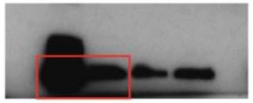
NUMB

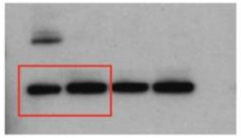
TSG101

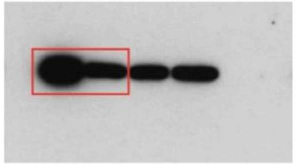
FLOT1

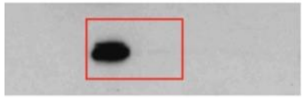
BCL2

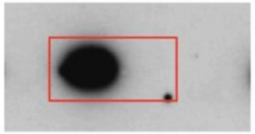
CYCS

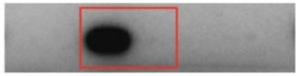
HSP90B1

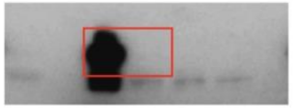
GOLGA2

Fig. S9D

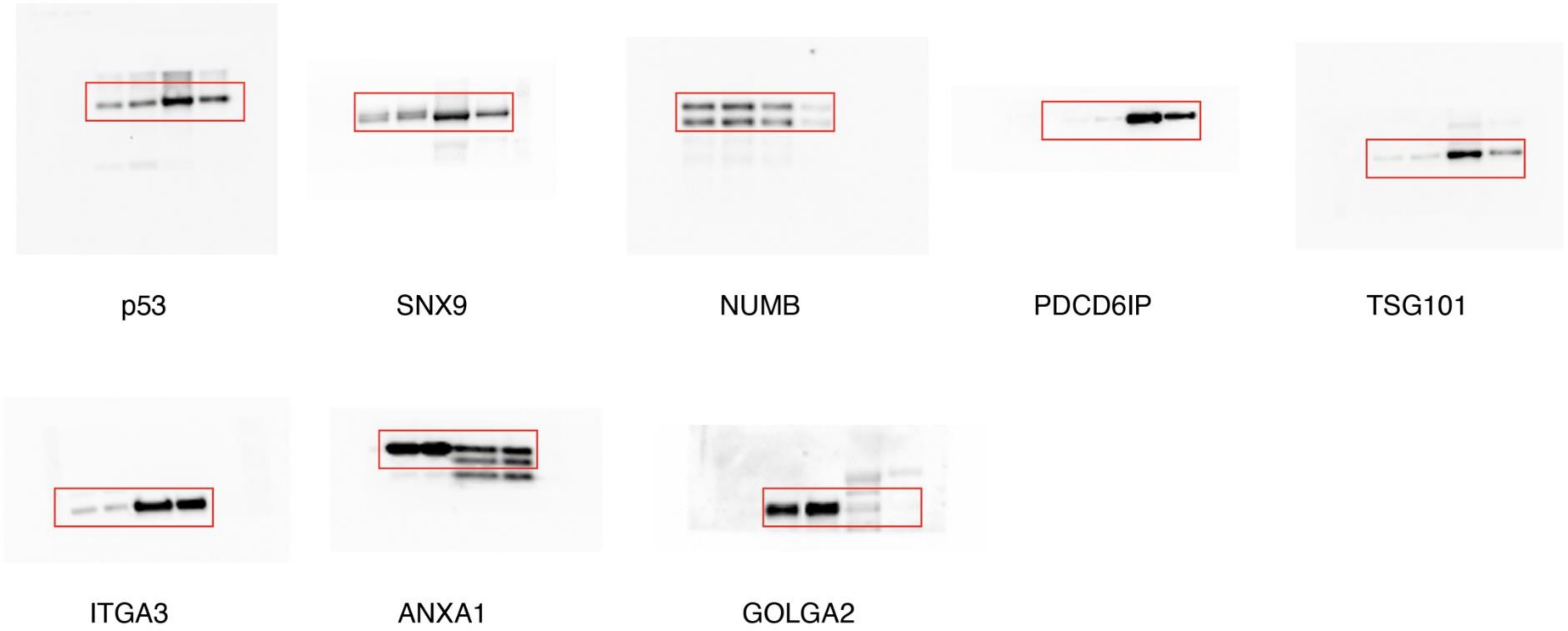

Fig. S9H

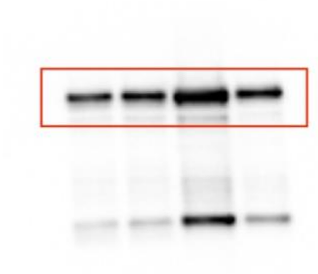

p53
(p53-GFP)

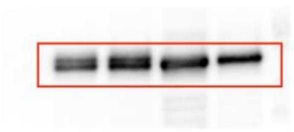

SNX9

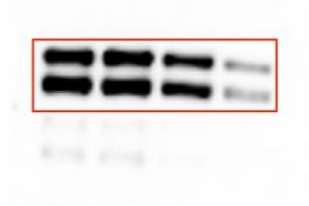

NUMB

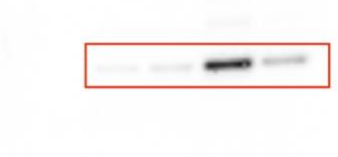

TSG101

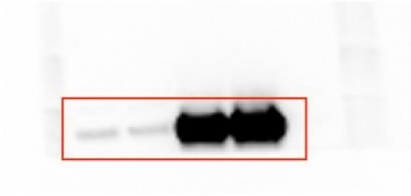

ITGA3

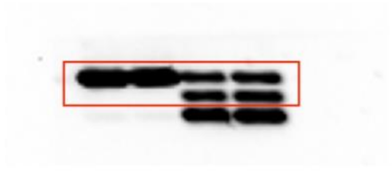

ANXA1

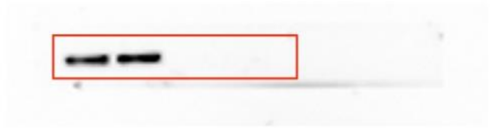

HSP90B1

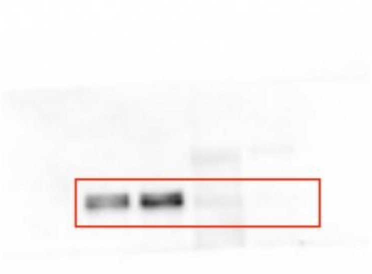

GOLGA2

Fig. S9J

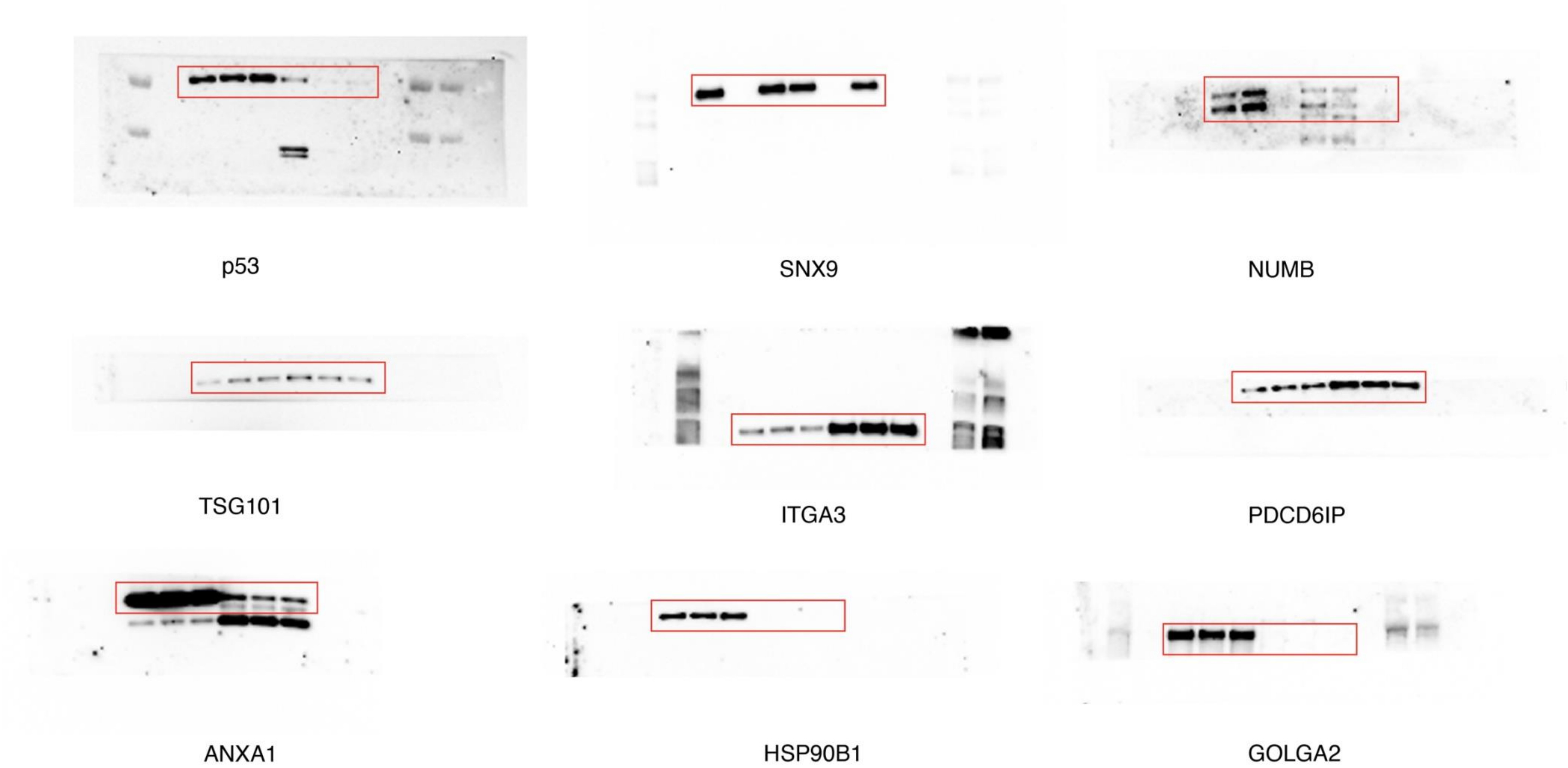

Fig. S9K

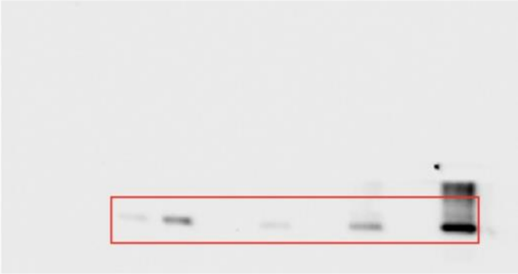

p53

Fig. S9L

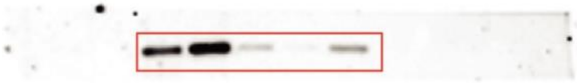

p53

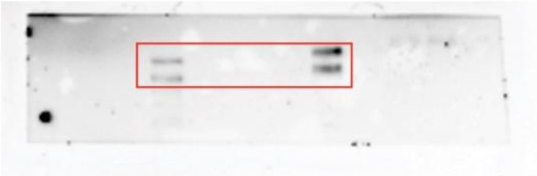

NUMB

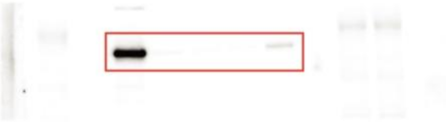

SNX9

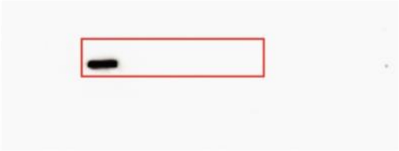

GAPDH

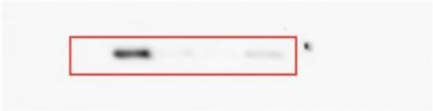

HNRNPA1

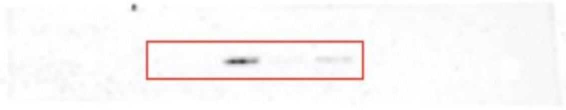

LMNB1

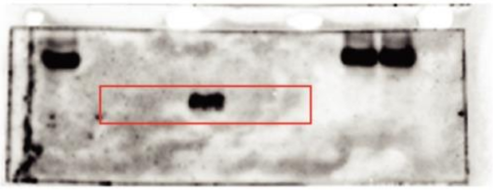

H3C13

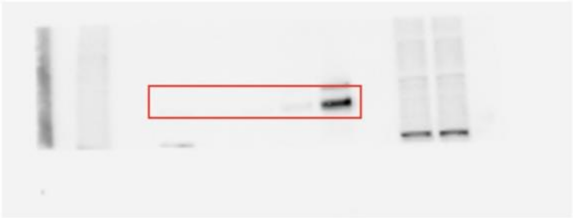

ITGA3

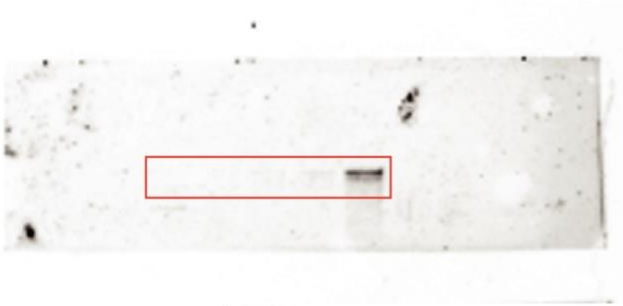

LRP6

Fig. S10A

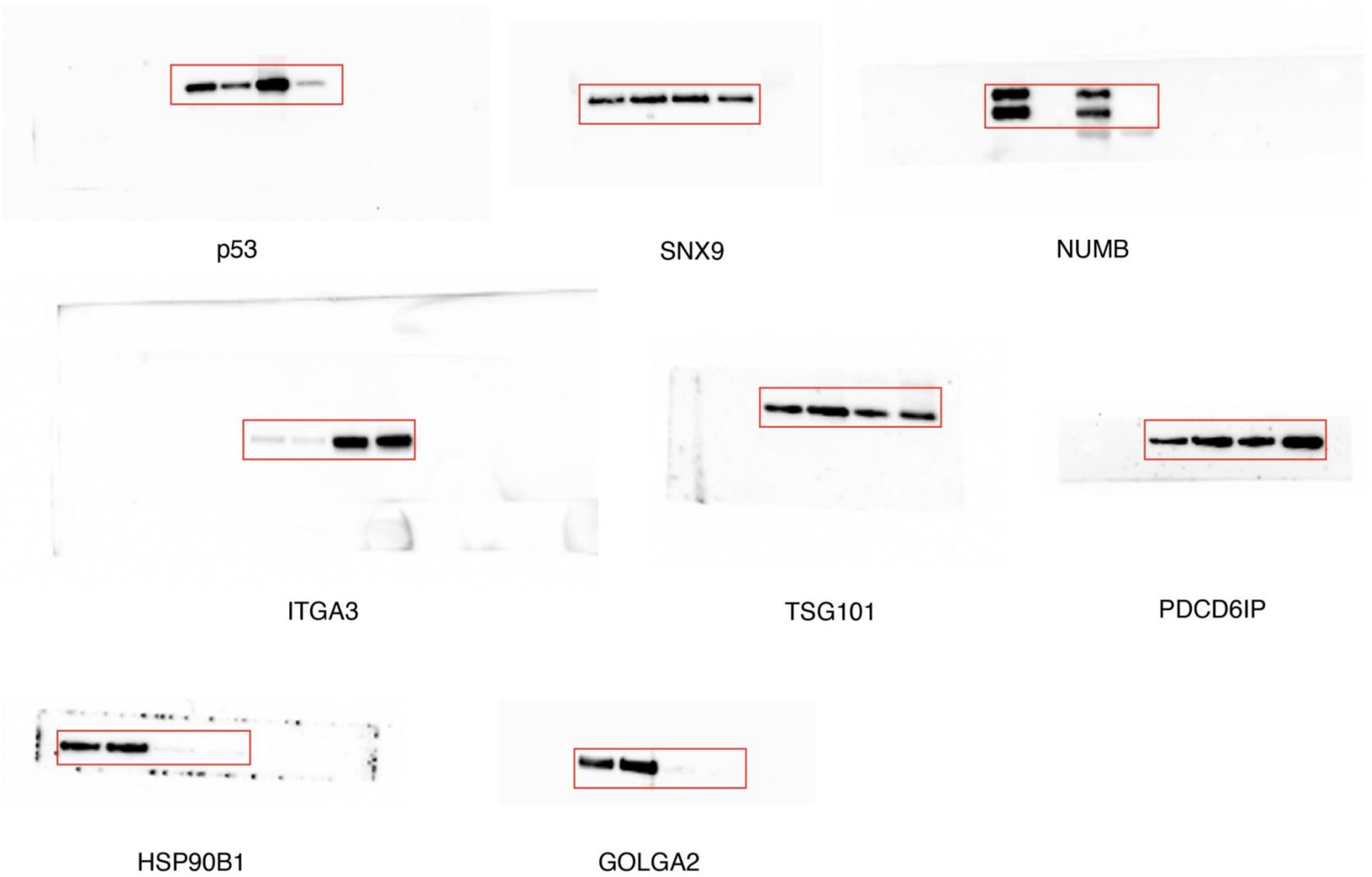

Fig. S10B

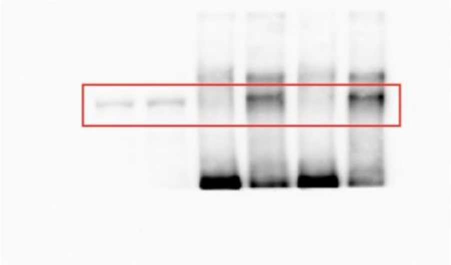

MDM2

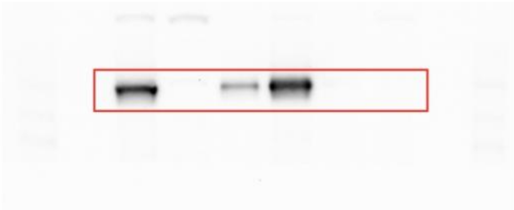

SNX9

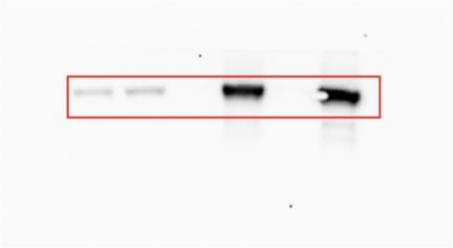

p53

Fig. S11A

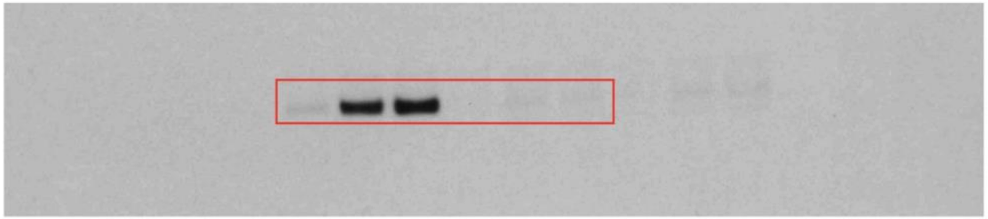

p53

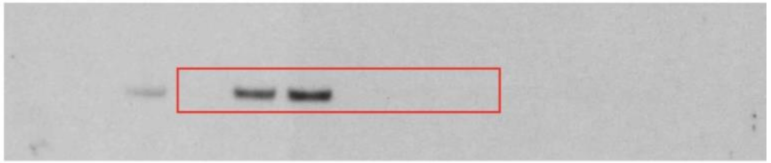

$p53^{ser15}$

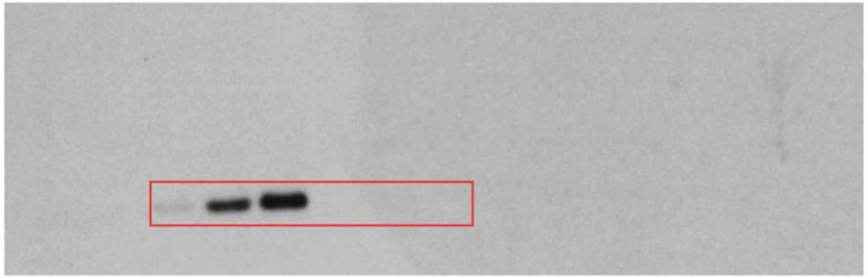

CDKN1A

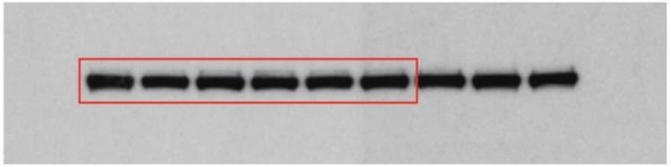

VCL
